# Natural Compounds from Hatikana Extract Potentiate Antidiabetic Actions as Displayed by In Vivo Assays and Verified by Network Pharmacological Tools

**DOI:** 10.1155/2021/6978450

**Published:** 2021-10-23

**Authors:** Md. Atiar Rahman, Md. Nazim Uddin, Nouf Abubakr Babteen, Afnan M. Alnajeebi, Zainul Amiruddin Zakaria, Salama Mostafa Aboelenin

**Affiliations:** ^1^Department of Biochemistry & Molecular Biology, University of Chittagong, Chittagong4331, Bangladesh; ^2^Institute of Food Science and Technology, Bangladesh Council of Scientific and Industrial Research, Dhaka 1205, Bangladesh; ^3^Department of Biochemistry, College of Science, University of Jeddah, Jeddah 80203, Saudi Arabia; ^4^Department of Biomedical Sciences, Faculty of Medicine and Health Sciences, Universiti Malaysia Sabah, (Jalan UMS), 88400 Kota Kinabalu, Sabah, Malaysia; ^5^Halal Product Development Unit, Halal Product Research Institute, Universiti Putra Malaysia, (UPM), 43400 Serdang, Selangor, Malaysia; ^6^Biology Department, Turabah University College, Taif University 21995, Saudi Arabia

## Abstract

**Background:**

Hatikana is a traditional medicinal plant used to treat inflammation, urolithiasis, goiter, cancer, wounds and sores, gastrointestinal, tumor, tetanus, arthritis, hepatic damage, neurodegeneration, and other ailments. The goal of this study is to investigate the antidiabetic properties of Hatikana extract (HKEx) and to construct the effects of its natural constituents on the genes and biochemical indices that are connected with them.

**Methods:**

HKEx was evaluated using GC-MS and undertaken for a three-week intervention in fructose-fed STZ-induced Wistar albino rats at the doses of HKEx50, HKEx100, and HKEx200 mg/kg bw. Following intervention, blood serum was examined for biochemical markers, and liver tissue was investigated for the mRNA expression of catalase (CAT), glutathione peroxidase (GPx), and superoxide dismutase (SOD1) by RTPCR analysis. Most abundant compounds (oleanolic acid, 7*α*, 28-olean diol, and stigmasterol) from GC-MS were chosen for the network pharmacological assay to verify function-specific gene-compound interactions using STITCH, STRING, GSEA, and Cytoscape plugin cytoHubba.

**Results:**

In vivo results showed a significant (*P* < 0.05) decrease of blood sugar, aspartate aminotransferase (AST), alanine aminotransferase (ALT), creatinine kinase (CK-MB), and lactate dehydrogenase (LDH) and increase of liver glycogen, glucose load, and serum insulin. Out of three antioxidative genes, catalase (*CAT*) and superoxide dismutase (*SOD1*) were found to be few fold increased. Oleanolic acid and stigmasterol were noticed to strongly interact with 27 target proteins. Oleanolic acid interacted with the proteins AKR1B10, CASP3, CASP8, CYP1A2, CYP1A2, HMGB1, NAMPT, NFE2L2, NQO1, PPARA, PTGIR, TOP1, TOP2A, UGT2B10, and UGT2B11 and stigmasterol with ABCA1, ABCG5, ABCG8, CTSE, HMGCR, IL10, CXCL8, NR1H2, NR1H3, SLCO1B1, SREBF2, and TNF. Protein-protein interaction (PPI) analysis revealed the involvement of 25 target proteins out of twenty seven. Cytoscape plugin cytoHubba identified TNF, CXCL8, CASP3, PPARA, SREBF2, and IL10 as top hub genes. Pathway analysis identified 31 KEGG metabolic, signaling, and immunogenic pathways associated with diabetes. Notable degree of PPI enrichment showed that SOD1 and CAT are responsible for controlling signaling networks and enriched pathways.

**Conclusion:**

The findings show that antioxidative genes have regulatory potential, allowing the HKEx to be employed as a possible antidiabetic source pending further validation.

## 1. Introduction

Diabetes mellitus (DM), a chronic metabolic disorder characterized by hyperglycemia resulting from increased hepatic glucose production, impaired insulin production by pancreatic *β* cells and insulin resistance [1]. Long-term impact of DM contributes to the damage of the renal, cardiovascular, endocrine and nervous tissues, and organs [2]. The use of plant-based medicines has recently been attracted much due to the unaffordability, noncompliance, and adverse effects of synthetic drugs. Network pharmacology has been used as a very powerful instrument to analyze the molecular pathways in order to fully determine the pharmacological effects of plant-derived drugs [3, 4]. This tool has drawn considerable interest to conduct systematic pharmacological studies on the relationship between biological processes and the treatment of herbal medicine. Indeed, network pharmacology methods were developed with the goal of prioritizing disease-associated genes, predicting the target profiles and pharmacological actions of herbal compounds, revealing drug-gene-disease comodule associations, screening synergistic multicompounds from herbal formulae in a high-throughput manner, and interpreting combinatorial rules and network regulations [5]. Studying the basic biological knowledge of plant-based drugs on the basis of network interaction will provide indepth insight or scientific evidence for the discovery of alternative medicine and enable us to understand their pharmacological function at the biomolecule stage [6]. The usefulness of network pharmacology has made it a dynamic tool for investigating the interactions between drugs and biological systems, including human organs, diseases, metabolic processes, and target proteins [7, 8]. Natural compounds from numerous plants have been approached for network pharmacology to be explored as new drugs in the current drug discovery method.

*Leea macrophylla*, locally named as Hatikana or Hostikorna, is an extraordinary medicinal plant widely distibuted in Bangladesh, India, Srilanka, Nepal, and China. Leaves and roots of *Leea macrophy* have been studied and so far evident as antioxidant [9], anti-inflammatory [10], antithrombotic [11] antilithiatic [12], and protective for goiter, gastric tumor, lipoma, and tetanus [13, 14]. In this study, the antidiabetic effect of *Leea macrophylla* extract (HKEx) has been evaluated using the animal model and the combination of pharmacology, and pharmacodynamics has been successfully used to clarify the in vivo effect at the stage of the molecular network [8].

## 2. Materials and Methods

### 2.1. Plant Material and Extraction

Hatikana samples (*Leea macrophylla* root samples) were collected from the cultivation plots of the Bangladesh Council of Science and Industrial Research (BCSIR), Rajshahi, in July 2015. Dr. Sheikh Bokhtear Uddin, a taxonomist and professor at the Department of Botany, University of Chittagong, identified the plant. The nomenclature of the plant has been confirmed from International Plant Name Index (IPNI) using http://powo.science.kew.org/. A sample specimen of the plant has been preserved in the institutional herbarium as an accession number SBU1109CTGUH for future reference. Hatikana samples were thoroughly washed to remove debris and earth remains followed by chopping into small pieces and shade-dried for about 15 days. Dried pieces were subsequently ground into powder using mechanical grinder (Miyako, Model No: DL-718, China). Resulting powder (800 g) was defatted by n-hexane (2.0 L) for two days of maceration with occasional stirring. The fat-free residue was further extracted with maceration in ethanol (99.9%) for 3 days, and collected supernatant was evaporated by using a rotary evaporator (RE 200, Bibby Sterling Ltd., UK) set at reduced pressure within 50-55°C. The concentrated crude extract of Hatikana (HKEx), semisolid dark-brown extract (w/W, 4.7%), was left in the air in petridish and allowed for a complete solvent evaporation and stored at 4°C for further use.

### 2.2. Phytochemical Analyses of the Extract

HKEx was assessed with GC-MS using the electron impact ionization (EI) process on a gas chromatograph (GC-17A, Shimadzu Corporation, Kyoto, Japan) coupled to a mass spectrometer (GC-MS TQ 8040, Shimadzu Corporation, Kyoto, Japan). A fused capillary column (Rxi-5 ms; 0.25 m film thickness, 0.32 mm internal diameter) coated with silica was used for the separation. The temperature of the inlet was set to 260°C, and the temperature of the oven was programmed to be 70°C (0 min); 10°C, 150°C (5 min); 12°C, 200°C (15 min); and 12°C, 220°C (5 min). At a constant pressure of 90 KPa, the column flow rate was 0.6 mL/min helium gas. The temperature of the auxiliary gas flow rate (GC to MS interface) was set at 280°C. With a scanning range of 40-350 amu, the MS was set in scan mode; the ionization mode was type EI (electron ionization). The mass range was set at the range of 50-550 m/z. For GC/MS analysis, the prepared one microliter (1 *μ*L) sample was injected and run in spiltless modes. The overall runtime of the GC-MS was 35 minutes. In the GC-MS library version NIST 08-S, all peak areas were compared to the database.

### 2.3. Experiments in the Animal Model

In an animal experiment, thirty (age: six-seven weeks, average body weight: 185.0 ± 5.0 g) fructose-fed (10% fructose solution, an appropriate concentration to induce partial pancreatic dysfunction by STZ) Wistar albino rats were randomly classified into normal control (NC, received distilled water), diabetes control (DBC, received STZ), and three treatment groups (HKEx at 50, 100, and 200 mg/kg bw). Following a week-long acclimatization in a confined setting (12-hour day-light cycle, room temperature 23 ± 2°C; humidity 60-65%), the animals were injected by a single dose of freshly prepared streptozotocin (60 mg/kg bw, dissolved in 0.1 M citrate buffer). After a week, animals with a nonfasting blood glucose level of around 300 mg/dL were considered to be diabetic and chosen for the experiment. Under the institutional ethical guidelines of the University of Chittagong (Faculty of Biological Sciences, AERB/CUBS/2019-05), the handling and treatment of the experimental animals were guaranteed.

### 2.4. Biochemical and Molecular Analyses of Collected Blood

At the third week of animal intervention, a single dose of glucose solution (2 g/kg body weight) was administered to assess the glucose tolerance capacity by measuring the subsequent blood glucose levels at 0, 30, 60, 90, and 120 min after administration by the tail prick method. At the end of the intervention, halothane anesthesia was used to sacrifice the animals, and blood and organs were collected. The entire blood, collected by cardiac puncture, was centrifuged for 15 min at 1100 g, and the separated serum was retained at -30°C for further analysis of lipid profile, aspartate transaminase (AST), alanine transaminase (ALT), creatinine kinase (CK-MB), lactate dehydrogenase (LDH), creatinine, and uric acids. Liver glycogen concentrations were measured by a phenol-sulfuric acid method as described by Lo et al. [15]. Total RNA extraction and mRNA expression of liver antioxidative enzymes: glutathione peroxidase, CAT, and SOD1 were accomplished using the protocol described by Mawa et al. [16]. Briefly, 500 mg of each liver sample was added shortly after dissection to 1 mL of TRIzol Reagent (Invitrogen, Ambion, USA) and homogenized with a tissue homogenizer (IKA, UltraTurrax, Germany). One mL of tissue homogenate was transferred to a microfuge tube for total RNA extraction by adding 0.2 mL chloroform and vigorously vortexed for 15 sec before incubating for 3 min at room temperature. The aqueous phase containing RNA was transferred to new tubes after centrifugation at 12,000 g for 15 min at 4°C, and the RNA was precipitated by mixing the aqueous phase with 0.5 mL isopropyl alcohol and incubating at room temperature for 10 min. After centrifuging the RNA pellets at 12,000 g for 10 min at 4°C, they were washed by mixing and vortexing with 1 mL 75 percent ethanol and recentrifuged at 7500 g for 5 min at 4°C. A Reverse Transcription System Kit (Promega) and aSimpliAMP Thermal Cycler were used to generate cDNA from 2 g of total RNA (Life Technologies, Applied Biosystem, USA). A Reverse Transcription System Kit (Promega) and aSimpliAMP Thermal Cycler were used to generate cDNA from 2 g of total RNA (Life Technologies, Applied Biosystem, USA). Briefly, total RNA was activated at 70°C for 10 min, and a 20 *μ*L reaction mix was made with 2.5 *μ*L MgCl_2_, 4 *μ*L of reverse transcription 10× buffer, 1 *μ*L of 10 mM dNTTP mixture, ribonuclease inhibitor (0.5 *μ*L), 1 *μ*L oligo DT, 1 *μ*L of GoScript reverse transcriptase enzyme, 1 ng RNA, and nuclease-free water to a final volume of 20 *μ*L. The reaction mixture was then incubated at 25°C for 5 min and 42°C for 60 min followed by incubation at 70°C for 15 min. For PCR amplification, the cDNA was diluted up to 50 l with nuclease-free water. The RT-qPCR was performed with a SYBR Green PCR Master Mix kit (Promega) and specific primers for the antioxidant enzymes-related genes: *CAT* and SOD1. Each 18 *μ*L reaction contained 9 *μ*L of master mix, 1.5 *μ*L of forward primer (10 *μ*M/liter), 1.5 *μ*L of reverse primer (10 *μ*M/liter), and 6 *μ*L of cDNA. The cycling parameters were as follows: initial incubation at 95°C for 3 min, 40 cycles of 95°C for 30 s, 51°C for 15 s, and 72°C for 30s, with a final extension at 72°C for 10 min. The specificity of the obtained products was confirmed by analysis of the amplified product dissociation curves. The data obtained were analyzed using the 2^-∆∆CT^ method. Within each sample, the target genes were then normalized to GAPDH.

### 2.5. Target Identification for the Computational Model

Three compounds oleanolic acid, stigmasterol, and 7 *α*, 28-olean diol from the list of GC-MS characterized compounds were selected for bioinformatic-based network pharmacological analyses. These compounds were preferred because they were also identified by other authors via vacuum liquid chromatography (VLC) analysis [17] from Hatikana as well as their abundance of occurrence in GC-MS analysis.

### 2.6. Bioactive Compound-Target Protein Network Construction

Target compounds for network pharmacology were selected from the GC-MS analysis data and the bioactive compounds isolated through vacuum liquid chromatography (VLC) for fractionation over silica gel (kieselgel 60H, 70-230 mesh) with a mobile phase of n-hexane: ethyl acetate [17]. STITCH 5 (http://stitch.embl.de/, ver.5.0) was used on the basis of network pharmacology-based prediction to classify target proteins associated with bioactive phytochemicals that were found in HKEx [18]. It calculates a score for each pair of protein-chemical interactions. Chemical names of bioactive compounds (oleanolic acid, NZ-15; 7 *α*, 28-olean diol, NZ-38; and stigmasterol, NZ-14) were put into STITCH 5 together to match their potential targets, with the organism selected as “Homo sapiens” and medium required interaction score being 0.4 (threshold level). Twenty-seven medium confidence target proteins were predicted, 15 for oleanolic acid (NZ-15), and 12 for stigmasterol (NZ-14), but no target was identified for the third compound, 7 alpha, 28-olean diol. The compound targets with no association with the interactions of compound proteins were not taken into account for further analysis. Compound-protein interaction data were imported into Cytoscape 3.6.1 software to compare and construct a compound-protein interaction network [19].

### 2.7. Construction of Protein-Protein Interaction (PPI) Network of the Predicted Genes

A PPI network of predicted genes was created through a search tool for the recovery of the interacting gene (STRING) database (https://string-db.org/cgi/input.pl; STRING-DB v11.0) [20]. The rank of the target proteins based on the degree of interactions in the PPI network was defined using the cytoHubba plugin Cytoscape [21]. The obtained protein interaction data of 27 target proteins were imported into Cytoscape 3.6.1 software to construct a PPI protein interaction network [22].

### 2.8. Gene Ontology (GO) and Kyoto Encyclopedia of Genomes and Genes (KEGG) Pathway Target Protein's Enrichment Analyses

Gene set enrichment analysis (GSEA) was used to identify the function of target proteins that interact with the active ingredients of LMR in gene function and signaling pathway [21]. The KEGG [22] pathways significantly associated with the predicted genes were identified. We analyzed the Gene Ontology (GO) function and KEGG pathway enrichment of proteins (25 interacted target proteins) involved in the PPI network. The target proteins involved in the molecular function (MF), cellular components (CC), biological process (BP), and the KEGG pathways were also described. The FDR value < 0.05, calculated by the Benjamini–Hochberg method [22], was considered as significant.

### 2.9. Statistical Analysis

Data are presented as a mean ± SD. Data analysis was made by SPSS (Statistical package for Social Science, Version 22.0) using one-way analysis of variance (ANOVA) followed by Tukey's multiple posthoc tests. At *P* < 0.05, the values were found to be significantly different.

## 3. Results

### 3.1. Primary Phytochemical Screening and GC-MS Analysis

**Figure 1:**
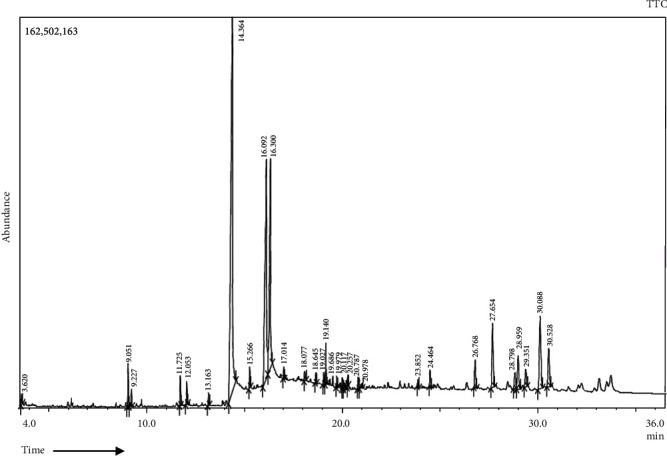
Gas chromatography-mass spectrometry profile of HKEx was obtained from GC-MS with the electron impact ionization (EI) method on a gas chromatograph (GC-17A, Shimadzu Corporation, Kyoto, Japan) coupled to a mass spectrometer (GC-MS TQ 8040, Shimadzu Corporation, Kyoto, Japan).

**Figure 2:**
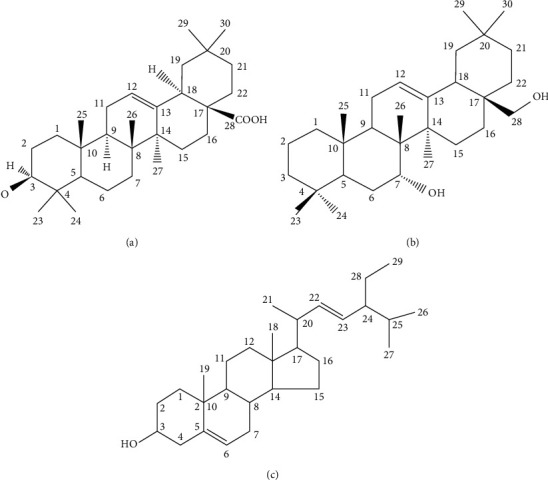
The three compounds (a) oleanolic acid, (b) 7*α*, 28-olean diol, and (c) stigmasterol have been isolated and identified by vacuum liquid chromatography (VLC) followed by other spectroscopic analyses.

Figure 1 provides an array of compounds (Table 1) obtained from the GC-MS analysis of HKEx. The constituents were identified by comparing their mass spectrum with those in the machine library and with genuine compounds. Oleanolic acid, stigmasterol, NZ-14 N-Hexadecanoic acid (37.15%), 7 *α*, 28-olean diol, 9-octadecenoic acid, octadecanoic acid (12.56%), *γ*-sitostenone (5.88%), 1,2,3-propanetriyl ester, (E, E, E)-(18.87%), and *γ*-Sitosterol (4.13%) were the main components. Among these compounds, three most abundant compounds (oleanolic acid, stigmasterol, and 7 *α*, 28-olean diol in Figure 2) were also identified by other authors via vacuum liquid chromatography (VLC) analysis [17].

### 3.2. Changes of Blood and Biochemical Parameters

**Figure 3:**
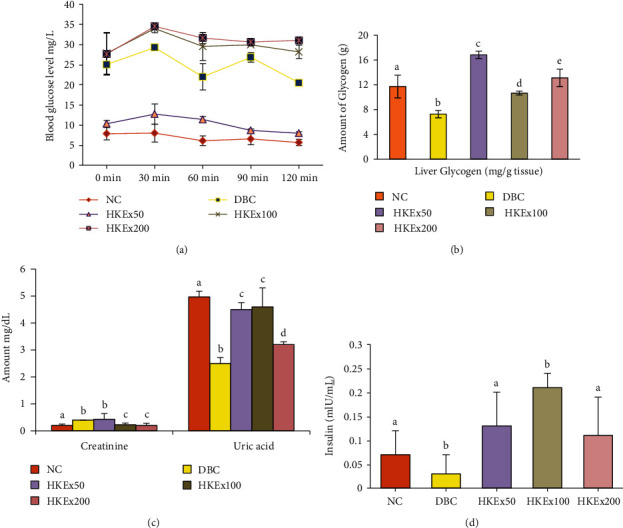
Effect of HKEx on (a) oral glucose tolerance, (b) liver glycogen, (c) serum creatinine and uric acid level, and (d) serum insulin levels in/after a three-week intervention of HKEx in albino rats (*n* = 6). Data are expressed as mean ± SD. All data were analyzed by statistical software SPSS (Statistical package for Social Science, IBM Corporation, NY, Version 22.0) followed by a Tukey's posthoc test for significance. *P* ≤ 0.05 was considered as significant.

Figure 3(a) demonstrates the oral glucose tolerance level of the experimental animals over the third week of intervention. The glucose tolerance ability of the lower dose (HKEx50) was significantly (*P* < 0.05) higher than the other groups including the diabetic control (DBC) group. The amount of liver glycogen present in experimental groups is shown in Figure 3(b). Liver glycogen in the HKEx-treated groups was significantly higher than in the DBC group, and even HKEx50 seemed to be more effective. Serum creatinine in the HKEx100 and HKEx200 was found to be significantly lower than in the DBC group as shown in Figure 3(c), but serum uric acid levels were increased in all the treatment groups (Figure 3(c)). The HKEx treatments resulted in a significant increase in insulin production compared to DBC groups (Figure 3(d)).

**Figure 4:**
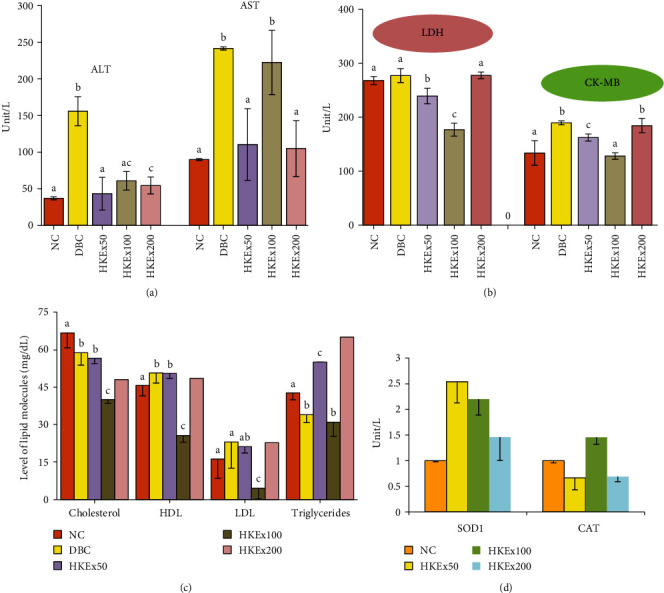
Effect of HKEx on the changes of (a) ALT-AST, (b) LDH-CKMB, (c) lipid profile, and (d) SOD1 and CAT mRNA expression in/after a three-week intervention in albino rats (*n* = 6). Data are expressed as mean ± SD. All data were analyzed by statistical software SPSS (Statistical package for Social Science, IBM Corporation, NY, Version 22.0) followed by a Tukey's posthoc test for significance.

Changes in serum alanine aminotransferase (ALT) and aspartate aminotransferase (AST) enzymes were summarized in Figure 4(a). All doses of HKEx were found to significantly reduce serum ALT levels while only HKEx50 and HKEx200 were able to decrease AST level significantly compared to DBC.  Figure 4(b) presents lactate dehydrogenase (LDH) and the heart-specific isozyme creatinine kinase (CK-MB) values. The serum LDH and CK-MB concentrations of the treatment groups HKEx50 and HKEx100 were significantly (*P* < 0.05) reduced compared to diabetic control animal.  Figure 4(c) displays the data for the serum lipid profile. The HDL (high-density lipoprotein) and LDL (low-density lipoprotein) of the treatment groups were significantly (*P* < 0.05) normalized by HKEx100. Triglyceride concentration was also found to be lowered in the treatment group (HKEx100).  Figure 4(d) illustrates the mRNA expression of genes encoded for biological enzymes SOD1 and CAT. In RT-qPCR, the mRNA expression of superoxide dismutase 1 (SOD1) was increased with all doses of HKEx. HKEx50 had a substantial (*P* < 0.005) elevation in the SOD1 mRNA expression among the treatment groups. HKEx100 was only able to upregulate the mRNA expression for CAT.

### 3.3. Analysis of Interactions with Active Ingredients and Target Proteins

**Figure 5:**
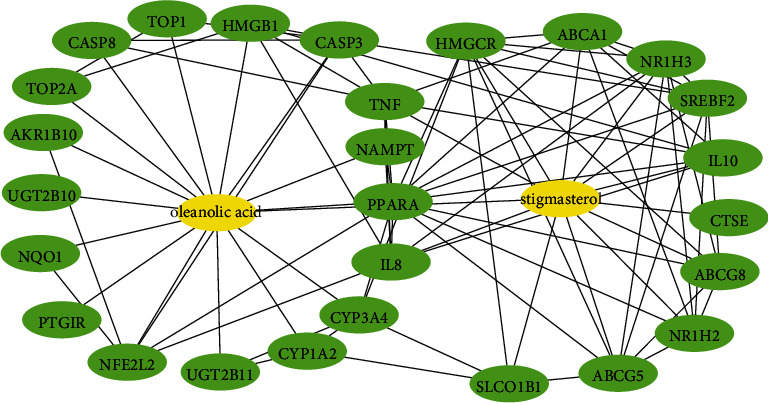
Network for compound-target interactions. The yellow node represents the compounds, and the blue node represents the top 27 target proteins. Stigmasterol interacts with eleven proteins while oleanolic acid interacted with sixteen proteins in compound-target protein interactions.

Antidiabetic mechanism of HKEx was evaluated through interactions of the target proteins with the functioning ingredients. We found that oleanolic acid (NZ-15) and stigmasterol (NZ-14) are significantly (PPI enrichment *P* value is 0.0001) interacted with 27 target proteins (Figure 5). Oleanolic acid interacted with AKR1B10, CASP3, CASP8, CYP1A2, CYP1A2, HMGB1, NAMPT, NFE2L2, NQO1, PPARA, PTGIR, TOP1, TOP2A, UGT2B10, and UGT2B11 and stigmasterol interacted with ABCA1, ABCG5, ABCG8, CTSE, HMGCR, IL10, CXCL8, NR1H2, NR1H3, SLCO1B1, SREBF2, and TNF. Interestingly, we found that oleanolic acid and stigmasterol also interact with each other in the compound targets interactions. Compounds and target protein interactions are displayed in Figure 5.

### 3.4. Construction and Analysis of Target Protein PPI Network

**Figure 6:**
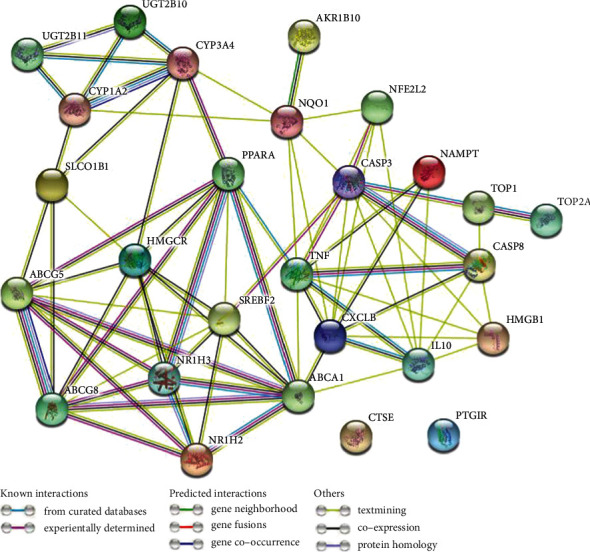
Protein–protein interaction (PPI) network of twenty seven target proteins. Only two target proteins (CTSE and PTGIR) are not involved in PPI. STRING database and Cytoscape plugin cytoHubba showed that twenty proteins are interacted in the PPI network.

PPI network plays substantial roles in the molecular processes, and abnormal PPI is the basis of many pathological conditions. Using the STRING database and Cytoscape plugin cytoHubba, all target proteins were mapped into the PPI network. Interestingly, we found that 25 target proteins are involved in PPI which have 77 edges, and average node degree 5.7 with the PPI enrichment *P* value was less than 1.0 × 10^−16^. This PPI network indicates that the greater the node degree, the better the relationship between the proteins which signify their role in the entire interaction network. Only CTSE and FTGIR proteins are not included in PPI. We got only one subnetwork in PPI, which included 25 target proteins (listed with degree of interaction in supplementary Table S1). Twenty five target proteins with top degree of interactions with other proteins illustrated in Figure 6 and in supplementary Table S1. ABCA1, TNF, CXCL8, CASP3, HMGCR, PPARA, SREBF2, ABCG8, ABCG8, ABCG5, and IL10 are the top ten hub proteins with maximum degree of interaction (Figure 6 and Supplementary Table S1).

### 3.5. GO Analysis of Interacted Target Proteins

**Figure 7:**
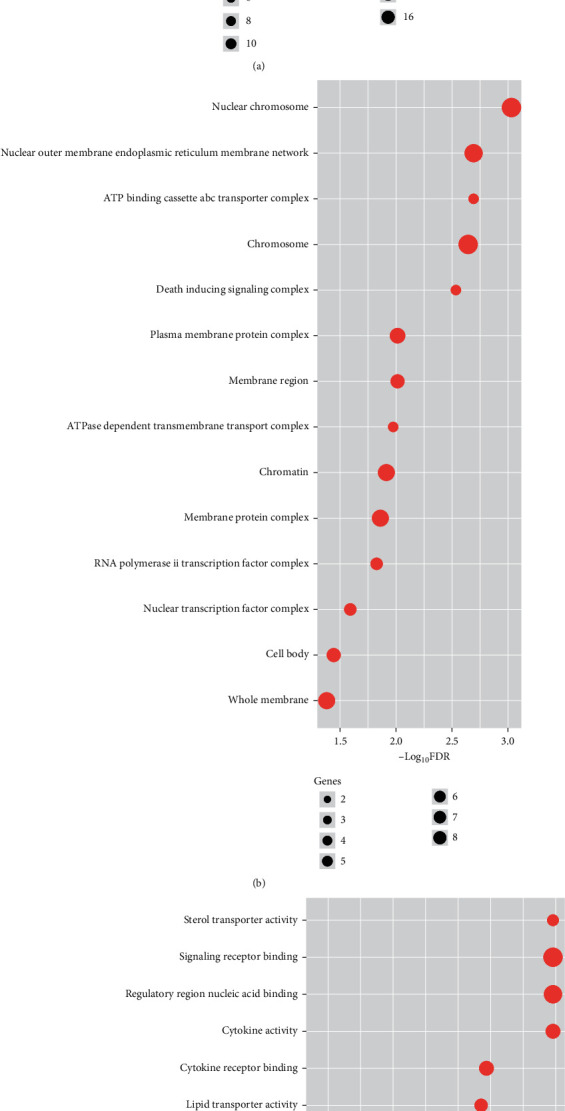
Gene Ontology (GO) enrichment analysis of the interacted target proteins. (a) Top 20 biological processes (BP). (b) 14 cellular components (CC). (c) Top 20 molecular functions (MF).

GO enrichment analysis of interacted target proteins (total 25) that interact with compounds was performed by GSEA. The top 20 significantly enriched terms in biological process (BP), 20 molecular functions (MF), and total 14 cellular components (CC) were selected, according to FDR value < 0.05. Significant BP was listed in supplementary Table S2, and top 20 BP are presented in Figure 7(a). Fourteen top identified CCs are illustrated in Figure 7(b) and supplementary Table S4. In molecular function (MF) category, top twenty molecular functions such as cytokine activity, cytokine receptor binding, and tumor necrosis factor receptor superfamily binding, lipid transporter activity, lipid transfer activity, and some other receptor activities are presented in Figure 7(c) and supplementary Table S3.

### 3.6. Target-Protein Set Enrichment Analysis of KEGG Pathways

**Figure 8:**
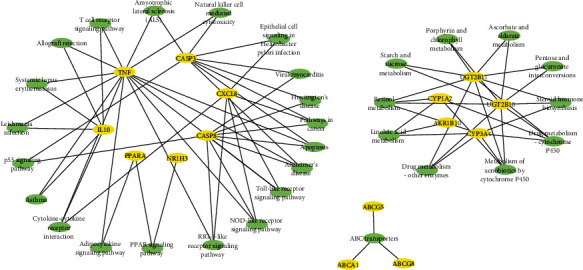
Network between enriched KEGG pathways and target proteins which are significantly interacted with compounds in this PPI network.

Link between the target proteins and the routes was further evaluated through identification of 31 KEGG pathways which were significantly associated with the target proteins (Figure 8 and Supplementary Table S5). These pathways were primarily involved in metabolism, immune regulation, neurological regulation, cellular development (apoptosis and p53 signaling pathway), cellular signaling, and PPAR signaling pathway.

### 3.7. Pathway-Associated Target Proteins Are Interacting with SOD1 and CAT

**Figure 9:**
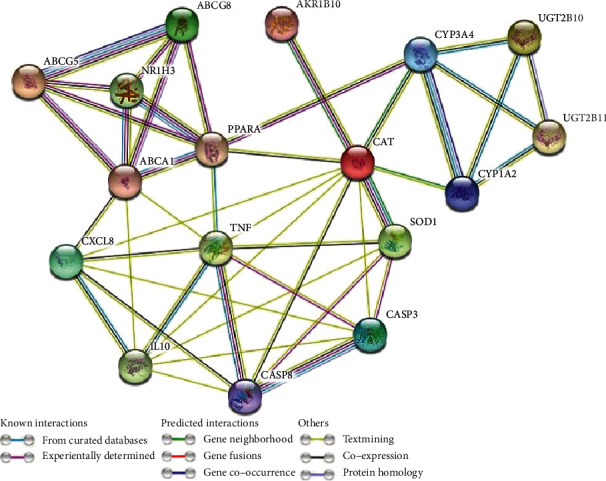
PPI Network between enriched KEGG pathway-associated target proteins and SOD1 and CAT.

We found that 15 target proteins (TNF, CXCL8, IL10, PPARA, CASP3, CASP8, AKR1B10, ABCA1, ABCG5, ABCG8, NR1H3, CYP3A4, CYP1A2, UGT2B11, and UGT2B10) are associated with the enrichment of pathways (Figure 9). The mRNAs of antioxidative enzymes SOD1 and CAT were found to increase multifold in animal intervention study. PPI interaction of SOD1, CAT, and pathway-associated target proteins was further investigated by using STRING tool and interestingly, 17 proteins were found to be associated with a single network with the PPI enrichment *P* value 6.6*e*-16 (Figure 9). CAT interaction degree was 10, and SOD1 interaction degree was 5.

## 4. Discussion

Plant-based therapies have long been used to treat T2DM due to their efficacy, affordability, and lack of adverse effects. This study [23] demonstrates the antidiabetic mechanism of a common antihyperglycemic plant combining both animal models and bioinformatic-based network pharmacology. Type 2 diabetes mellitus is a metabolic derangement with abnormal reaction to insulin and *β*-cell dysfunction of the pancreas leading to unexplained weight loss, energy balance changes, retinopathy, neuropathy, nephropathy, and vascular complications. This study evaluated the effects of HKEx on the involvement of SOD1 and CAT antioxidative enzymes and associated genes via a network pharmacological evaluation.

In this research, streptozotocin was chosen to induce diabetes, because its alkylating properties directly target pancreatic *β*-cells to damage [21]. Because of the structural similarity with glucose, selectivity for *β*-cells is correlated with preferential accumulation of streptozotocin in *β*-cells after entry through GLUT-2 glucose transporter receptors [24]. As part of drug discovery, plants have always been good source while many of the currently available drugs have been directly or indirectly derived from them. Antidiabetic action has been found in a wide range of plant-derived active principles. In that light, HKEx containing number of phytoconstituents could attribute to the decrease of blood glucose levels in animal model. In particular, the glucose tolerance capacity of the extract was noted at the lower dose of HKEx50 mg/kg during the third week of intervention. Moreover, liver glycogen levels were found substantially higher in the treatment groups than in the DBC group. The liver is essential for maintaining appropriate glucose homeostasis, as it delivers glucose during fasting and stores glucose afterward. However, these hepatic processes are dysregulated in diabetes mellitus, and this imbalance contributes to hyperglycemia in the fasted and postprandial states which is consistent with the decreased glycogen level in the liver of the DBC group [25]. The significant decrease of creatinine levels is an indication of protective effect of HKEx in diabetic nephropathy. Stimulation of insulin secretion by plant extract is documented by many researchers [26] who have noticed the potentiation of glucose-induced insulin release from isolate mouse islets. Our results are consistent to increase the insulin secretion by two of the doses.

Insulin resistance, on the other hand, results in hyperinsulinemia, which is responsible for dyslipidemia, increased sensitivity of LDL to oxidation, and decreased tolerance to glucose [27]. In several recent studies, serum ALT and AST have been found to be increased due to liver damage, and serum LDH and CK-MB have been increased due to cardiac damage in diabetic conditions, called diabetic cardiomyopathy [26, 28, 29]. Hyperglycemia is also associated with insulin resistance in T2DM and is a highly independent cardiovascular disease indicator. In treatment groups, the decrease in the CK-MB cardiac marker suggests that the extract could be showing a successful activity to boost cardiac conditions [30]. The information suggests that HKEx has a protective effect on diabetes-related heart damage. Serum ALT, AST, LDH, and CK-MB elevation in DBC appear to be due to STZ-induced cellular damage while ALT and AST were found to be substantially recovered by HKEx treatments implying the increased activity of liver and liver aminotransferase. Lipid homeostasis in this research is mainly achieved through the increase of HDL and decrease of LDL. This effect of HKEx might be attributable to the reduced synthesis of fatty acids and other cholesterol derivatives [31].

The major phytochemical constituents in addition to the three target compounds oleanolic acid, 7 *α*, 28-olean diol, and stigmasterol for network pharmacology, some fatty acids appear to be major more prevalent compounds in GC-MS analyses. The role of saturated fatty acids in the control of anti-inflammatory pathways has been justified by previous investigations. N-Hexadecanoic acid is literally displayed as antioxidant, anti-inflammatory, and hypocholesterolemic. The enzyme kinetic analysis showed that n-hexadecanoic acid acted as a competitive phospholipase A2 inhibitor and controlled inflammatory cytokine synthesis by controlling the release of arachidonic acid [32], octadecanoic acid and 9-octadecenoic acid, 1, 2, 3-propanetriyl ester, (E, E, E)-exercise antioxidants, immunomodulators, and immunostimulants [33]. Again, by growing the activity of Cu/Zn SOD and CAT in cultured cortical neurons, octadecanoic acid (stearic acid) exhibits defense against oxidative stress by substantially decreasing H2O2 treatment by 24 h and thus exerting neuroprotective effects [34]. The levels of glutathione, SOD1, and CAT are significantly increased by phytosterol and stigmasterol [35] which is consistent with our observation.

Antioxidant effects are generally known to be the most effective way to protect the number of diseases including diabetes. This plant was evident in promising in vitro antioxidant potentials that were confirmed with certain antioxidant enzymes' gene expression profile. Interestingly, however, the administration of HKEx to the STZ-induced animals upregulated the expression profile of these genes, implying that an antioxidant-dependent event mediated the pancreatic protective effects of HKEx. In fact, the twofold increase in the SOD1 expression observed was remarkable because it could imply a very rapid HKEx-induced antioxidative response. Similar findings have been recorded with other extracts attributed to the potent phytochemicals of antioxidants, as observed in HKEx.

The in vitro and in vivo findings have been verified using network pharmacological study which is currently facing a great development opportunities and challenges in terms of theoretical analyses, algorithm development, and applications in the Big Data era, but the challenge of merging enormous clinical and experimental data with scientific verification to expose the regulatory mechanisms of network pharmacology in order to conduct research more effectively has become a major concern for researchers. Therefore, data validation before clinical application is an inevitable step to be followed to avoid ambiguous use of network pharmacology [36]. Therefore, an experimental validation needs to be warranted before applying these findings into clinical application. PPI network plays substantial roles in molecular processes, and abnormal PPI is the basis of many pathological conditions [37]. From the PPI of targeted protein, we identified hub nodes which are markedly associated with diabetes. For example, the 10-degree interaction protein ABCA1 plays an important role in controlling lipid metabolism, and a defect in this gene interferes with the lipid transport of HDL cholesterol associated with the production of T2DM [38]. Peripheral insulin resistance and elevated plasma glucose and insulin levels are correlated with TNF-alpha concentration prior to the onset of T2DM [39]. In male mice with diabetes, the CXCL8 antagonist is evident to enhance diabetic nephropathy and attenuate high glucose-induced mesangial injury [40]. Caspase-3 promotes diabetic kidney disease [41]. It was stated that the HMGCR gene in population studies was associated with higher risk of T2DM [42]. It is proposed that interleukin-10 (IL10), an anti-inflammatory cytokine, has a protective role in T2D [43]. Our results shows that the immunological target proteins including IL10, CXCL8, and TNF are located in the PPI networks with top degree of interaction, indicating that this PPI network is associated with immunological activities. Human chemokines have been shown to be associated with or involved in the pathogenesis of type 1 diabetes [44].

In addition, we identified GO pathway enrichment analysis of target protein. The GO analysis indicating that the target proteins may bind with plasma membrane, chromosome, chromatin, regulatory region nucleic acid, or/and cellular receptor of cells for mediating the process of metabolic, immunological, signaling, and/or other activities, so as to exert signaling and antidiabetic potential of compounds. Pathway analysis revealed some pathways which are also associated with diabetes. Retinol metabolism is the most significantly (FDR < 8.6 × 10^−6^) enriched pathway. Retinoids and retinoid-related proteins involved with signaling molecules linking obesity with the production of type II diabetes and in pancreatic *β*-cell biology/insulin secretion [45]. Linoleic acid metabolism is metabolism is another enriched pathway, and it was indicated that the prevention of T2DM should include increased linoleic acid consumption [46]. Metabolic pathway research showed that possible metabolic pathways are primarily involved in the pathway of interconversion of pentose and glucoronate and the metabolism of starch and sucrose, which changed specifically in the T2D environment [47]. PPAR signaling pathway and adipocytokine signaling pathway are also dysregulated in T2DM [21, 48, 49]. Moreover, we found that CAT and SOD1 are controlling the PPI network of target proteins which are significantly associated with enriched pathways, suggesting that these two genes are responsible for controlling this signaling networks and enriched pathways. Altogether, it indicates that the target proteins are associated with the alteration or dysregulation of diabetic pathways and the phytoconstituents *of L. macrophylla* are highly prospective to be processed further for befitted uses in diabetes.

KEGG pathways are primarily involved in metabolism (retinol metabolism, cytochrome P450 xenobiotic metabolism, cytochrome metabolism of drugs, linoleic acid metabolism, drug metabolism, other enzymes, biosynthesis of steroid hormones, ascorbate and aldarate metabolism, interconversion of pentose and glucuronate, porphyrin and chlorophyll metabolism, and starch and sucrose metabolism), immune regulation (cytokine-cytokine receptor interaction, T cell receptor signaling pathway, natural killer cell-mediated cytotoxicity, adipocytokine signaling pathway, Toll-like receptor signaling pathway, allograft rejection, and systemic lupus erythematosus), neurological regulation (amyotrophic lateral sclerosis), cellular development (apoptosis and p53 signaling pathway), cellular signaling (NOD-like receptor signaling pathway, RIG-I-like receptor signaling pathway, and PPAR signaling pathway), molecular transportation (ABC transporters), and retinol metabolism linoleic acid consumption, interconversion of pentose and glucuronate metabolism [50] and PPAR signaling pathway [51–53]. Our anticipated SOD1 and CAT genes are very likely to influence the networks of pathways because all 17 proteins for PPI interaction of SOD1, CAT, and pathway-associated target proteins used in STRING tool were found to be associated with a single network with the PPI enrichment *P* value 6.6*e*-16. Importantly, CAT interaction degree 10 and SOD1 interaction degree 5 in the PPI network suggest that these two genes are responsible for controlling this signaling networks and enriched pathways. Based on the compound–protein target relationships, it is evident that compounds are acting on the multitarget genes and proteins which are substantial contributions of HKEx on biological and physiological activities to manage diabetes.

## 5. Conclusions

This research has meaningfully justified the use of L. macrophylla in T2DM which is a multigene-metabolic complication pivotalizing the oxidative stress. Network pharmacological tools successfully bridged the role of bioactive compounds from L. macrophylla in controlling different metabolic and molecular pathways including the upregulation of most prominent biological antioxidative enzymes SOD1 and CAT. This worthy piece of work can lead to unveil the prospects of this plant material through justifying other formulatory necessities.

## Figures and Tables

**Table 1:** Compounds identified in the ethanol extract of HKEx by GC-MS.

S.N	Name of the compound	RT	Peak area (%)
1.	2,2-Bis (chloromethyl)-1-propanol	3.620	0.43
2.	2H-Pyran-2-one, tetrahydro-4-hydroxy-6-pentyl-	9.051	1.36
3.	Butylated hydroxytoluene	9.227	0.59
4.	Benzaldehyde, 3-ethoxy-	11.725	0.99
5.	Tetradecanoic acid	12.053	0.76
6.	Pentadecanoic acid	13.163	0.44
7.	n-Hexadecanoic acid	14.364	37.15
8.	l-(+)-ascorbic acid 2,6-dihexadecanoate	15.266	0.72
9.	9-Octadecenoic acid, 1,2,3-propanetriyl ester, (E,E,E)-	16.092	18.87
10.	Octadecanoic acid	16.300	12.56
11.	12,13-Epoxy-octadec-9-enoic acid, DMOX derivative	17.014	0.38
12.	Eicosanoic acid	18.077	0.45
13.	Docosanal	18.645	0.32
14.	(2,3-Diphenylcyclopropyl)methyl phenyl sulfoxide, trans-	19.027	0.45
15.	2-Hydroxy-4-methoxy-7-methyl-7,8,9,10,11,12,13,14-octahydro-6-oxabenzocyclododecen-5-one	19.140	1.40
16.	Bis(2-ethylhexyl) phthalate	19.686	0.32
17.	(2,3-Diphenylcyclopropyl)methyl phenyl sulfoxide, trans-	19.979	0.42
18.	(2,3-Diphenylcyclopropyl)methyl phenyl sulfoxide, trans-	20.117	0.52
19.	7-Methoxy-3-(3,4-dimethoxyphenyl)-4H-chromen-4-one	20.257	0.35
20.	Tetrapentacontane, 1,54-dibromo-	20.787	0.63
21.	2,2-Dimethyl-6-methylene-1-[3,5-dihydroxy-1-pentenyl]cyclohexan-1-perhydrol	20.978	0.31
22.	Stigmasta-4,7,22-trien-3.Beta.-ol	23.852	0.29
23.	Oleanolic acid	24.464	0.72
24.	Stigmasterol	26.768	1.58
25.	*γ*-Sitosterol	27.654	4.13
26.	7*α*, 28-Olean diol	28.798	1.15
27.	4,22-Cholestadien-3-one	28.959	2.64
28.	Cyclopropa[5,6]-33-norgorgostan-3-ol, 3',6-dihydro-, (3.Beta.,5.Beta.,6.Alpha.,22.Xi.,23.Xi.)-	29.351	1.21
29.	*γ*-Sitostenone	30.088	5.88

## Data Availability

Data used in this sutdy are included in the article and rest of them are reposited as Supplementary Materials.

## Supplementary Materials

Supplementary Materials The following materials are available in the journal's online system: table S1: list of target proteins with degree of interactions in PPI network. Table S2: list of biological process. Table S3: list of molecular functions. Table S4: list of cellular components. Table S5: list of KEGG pathways.
