# Supplementary material for: Natural Compounds from Hatikana Extract Potentiate Antidiabetic Actions as Displayed by In Vivo Assays and Verified by Network Pharmacological Tools
Source: Biomed Res Int. 2021 Oct 23;2021:6978450. doi: 10.1155/2021/6978450 (PMC8557063; doi:10.1155/2021/6978450)
Supplement: Supplementary Materials — The following materials are available in the journal's online system: table S1: list of target proteins with degree of interactions in PPI network. Table S2: list of biological process. Table S3: list of molecular functions. Table S4: list of cellular components. Table S5: list of KEGG pathways. [file 6978450.f1.docx]

**Original article**

**Natural compounds from Hatikana extract potentiate antidiabetic actions as displayed by *in vivo* assays and verified by network-pharmacological tools**

Md. Atiar Rahman^1^*, Md. Nazim Uddin^2^, Nouf Abubakr Babteen^3^, Afnan M. Alnajeebi^3^, Zainul Amiruddin Zakaria*^4^

*^1^Department of Biochemistry & Molecular Biology, University of Chittagong, Chittagong-4331, Bangladesh*

*^2^Institute of Food Science and Technology, Bangladesh Council of Scientific and Industrial Research, Dhaka 1205, Bangladesh.*

*^3^Department of Biochemistry, Collage of Science, University of Jeddah, Jeddah*- 80203*, Saudi Arabia*

*^4^Department of Biomedical Science, Faculty of Medicine and Health Sciences, Universiti Putra Malaysia, UPM Serdang 43400, Selangor, Malaysia,*

**Running title**: Antidiabetic Network pharmacology for *Leea macrophylla*

**Correspondence:** Md. Atiar Rahman PhD, Professor, Department of Biochemistry & Molecular Biology, University of Chittagong, Chittagong-4331, Bangladesh, Tel: +88-031-2606001-10, Extension- 4334, Fax: +88-031-726310, E-mail:atiar@cu.ac.bd

and

Zainul Amiruddin Zakaria, Professor, Department of Biomedical Science, Faculty of Medicine and Health Sciences, Universiti Putra Malaysia, UPM Serdang 43400, Selangor, Malaysia, Email: zaz@upm.edu.my

**Supplementary Table S1. List of target proteins with degree of interactions in PPI network**

| **Rank** | **Name** | **Degree of interaction number** |
| --- | --- | --- |
| 1 | ABCA1 | 10 |
| 1 | TNF | 10 |
| 3 | CXCL8 | 9 |
| 3 | CASP3 | 9 |
| 3 | HMGCR | 9 |
| 6 | PPARA | 8 |
| 6 | SREBF2 | 8 |
| 6 | ABCG8 | 8 |
| 6 | ABCG5 | 8 |
| 6 | IL10 | 8 |
| 11 | NR1H3 | 7 |
| 11 | CYP3A4 | 7 |
| 11 | NQO1 | 7 |
| 14 | CASP8 | 6 |
| 14 | NR1H2 | 6 |
| 16 | SLCO1B1 | 5 |
| 16 | HMGB1 | 5 |
| 16 | CYP1A2 | 5 |
| 16 | NFE2L2 | 5 |
| 20 | TOP1 | 3 |
| 20 | UGT2B11 | 3 |
| 20 | UGT2B10 | 3 |
| 20 | NAMPT | 3 |
| 24 | AKR1B10 | 1 |
| 24 | TOP2A | 1 |

| **Supplementary Table S2. List of biological process** | | | | |
| --- | --- | --- | --- | --- |
| **Biological process** | **Description** | **Genes in Overlap** | **p-value** | **FDR q-value** |
| GO_RESPONSE_TO_DRUG | Any process that results in a change in state or activity of a cell or an organism (in terms of movement, secretion, enzyme production, gene expression, etc.) as a result of a drug stimulus. A drug is a substance used in the diagnosis, treatment or prevention of a disease. [GOC:jl] | 15 | 9.55E-18 | 7.19E-14 |
| GO_NEGATIVE_REGULATION_OF_LIPID_LOCALIZATION | Any process that stops, prevents or reduces the frequency, rate or extent of lipid localization. [GO_REF:0000058, GOC:TermGenie, PMID:17564681] | 8 | 3.50E-17 | 1.32E-13 |
| GO_RESPONSE_TO_OXYGEN_CONTAINING_COMPOUND | Any process that results in a change in state or activity of a cell or an organism (in terms of movement, secretion, enzyme production, gene expression, etc.) as a result of an oxygen-containing compound stimulus. [GOC:pr, GOC:TermGenie] | 16 | 2.79E-16 | 7.00E-13 |
| GO_RESPONSE_TO_LIPID | Any process that results in a change in state or activity of a cell or an organism (in terms of movement, secretion, enzyme production, gene expression, etc.) as a result of a lipid stimulus. [GOC:sl] | 13 | 3.60E-15 | 6.79E-12 |
| GO_RESPONSE_TO_MOLECULE_OF_BACTERIAL_ORIGIN | Any process that results in a change in state or activity of an organism (in terms of movement, secretion, enzyme production, gene expression, etc.) as a result of a stimulus by molecules of bacterial origin such as peptides derived from bacterial flagellin. [GOC:rl, GOC:sm] | 10 | 1.26E-14 | 1.90E-11 |
| GO_REGULATION_OF_LIPID_LOCALIZATION | Any process that modulates the frequency, rate or extent of lipid localization. [GO_REF:0000058, GOC:TermGenie, PMID:17564681] | 8 | 6.99E-14 | 8.78E-11 |
| GO_RESPONSE_TO_ENDOGENOUS_STIMULUS | Any process that results in a change in state or activity of a cell or an organism (in terms of movement, secretion, enzyme production, gene expression, etc.) as a result of a stimulus arising within the organism. [GOC:sm] | 14 | 3.50E-13 | 3.77E-10 |
| GO_REGULATION_OF_LIPID_STORAGE | Any process that modulates the rate, frequency or extent of lipid storage. Lipid storage is the accumulation and maintenance in cells or tissues of lipids, compounds soluble in organic solvents but insoluble or sparingly soluble in aqueous solvents. Lipid reserves can be accumulated during early developmental stages for mobilization and utilization at later stages of development. [GOC:BHF, GOC:dph, GOC:tb] | 6 | 4.20E-13 | 3.95E-10 |
| GO_CHOLESTEROL_STORAGE | The accumulation and maintenance in cells or tissues of cholesterol, cholest-5-en-3 beta-ol, the principal sterol of vertebrates and the precursor of many steroids, including bile acids and steroid hormones. [GOC:BHF, GOC:dph, GOC:tb] | 5 | 8.82E-13 | 7.38E-10 |
| GO_NEGATIVE_REGULATION_OF_LIPID_STORAGE | Any process that decreases the rate, frequency or extent of lipid storage. Lipid storage is the accumulation and maintenance in cells or tissues of lipids, compounds soluble in organic solvents but insoluble or sparingly soluble in aqueous solvents. Lipid reserves can be accumulated during early developmental stages for mobilization and utilization at later stages of development. [GOC:BHF, GOC:dph, GOC:tb] | 5 | 1.18E-12 | 8.85E-10 |
| GO_CHOLESTEROL_EFFLUX | The directed movement of cholesterol, cholest-5-en-3-beta-ol, out of a cell or organelle. [GOC:sart] | 6 | 1.57E-12 | 1.05E-09 |
| GO_REGULATION_OF_LIPID_TRANSPORT | Any process that modulates the frequency, rate or extent of the directed movement of lipids into, out of or within a cell, or between cells, by means of some agent such as a transporter or pore. [GOC:mah] | 7 | 1.67E-12 | 1.05E-09 |
| GO_REGULATION_OF_STEROL_TRANSPORT | Any process that modulates the frequency, rate or extent of the directed movement of sterols into, out of or within a cell, or between cells, by means of some agent such as a transporter or pore. [GOC:mah] | 6 | 2.91E-12 | 1.68E-09 |
| GO_CELLULAR_RESPONSE_TO_OXYGEN_CONTAINING_COMPOUND | Any process that results in a change in state or activity of a cell (in terms of movement, secretion, enzyme production, gene expression, etc.) as a result of an oxygen-containing compound stimulus. [GOC:pr, GOC:TermGenie] | 12 | 3.17E-12 | 1.69E-09 |
| GO_LIPID_LOCALIZATION | Any process in which a lipid is transported to, or maintained in, a specific location. [GOC:BHF, GOC:dph, GOC:tb] | 9 | 3.37E-12 | 1.69E-09 |
| GO_LIPID_STORAGE | The accumulation and maintenance in cells or tissues of lipids, compounds soluble in organic solvents but insoluble or sparingly soluble in aqueous solvents. Lipid reserves can be accumulated during early developmental stages for mobilization and utilization at later stages of development. [GOC:dph, GOC:mah, GOC:tb, PMID:11102830] | 6 | 5.07E-12 | 2.39E-09 |
| GO_RESPONSE_TO_ORGANIC_CYCLIC_COMPOUND | Any process that results in a change in state or activity of a cell or an organism (in terms of movement, secretion, enzyme production, gene expression, etc.) as a result of an organic cyclic compound stimulus. [GOC:ef] | 11 | 8.41E-12 | 3.73E-09 |
| GO_RESPONSE_TO_BACTERIUM | Any process that results in a change in state or activity of a cell or an organism (in terms of movement, secretion, enzyme production, gene expression, etc.) as a result of a stimulus from a bacterium. [GOC:hb] | 10 | 1.13E-11 | 4.73E-09 |
| GO_NEGATIVE_REGULATION_OF_CHOLESTEROL_STORAGE | Any process that decreases the rate or extent of cholesterol storage. Cholesterol storage is the accumulation and maintenance in cells or tissues of cholesterol, cholest-5-en-3 beta-ol, the principal sterol of vertebrates and the precursor of many steroids, including bile acids and steroid hormones. [GOC:BHF, GOC:dph, GOC:tb] | 4 | 1.76E-11 | 6.91E-09 |
| GO_LIPID_METABOLIC_PROCESS | The chemical reactions and pathways involving lipids, compounds soluble in an organic solvent but not, or sparingly, in an aqueous solvent. Includes fatty acids; neutral fats, other fatty-acid esters, and soaps; long-chain (fatty) alcohols and waxes; sphingoids and other long-chain bases; glycolipids, phospholipids and sphingolipids; and carotenes, polyprenols, sterols, terpenes and other isoprenoids. [GOC:ma] | 12 | 1.84E-11 | 6.91E-09 |
| GO_RESPONSE_TO_EXTRACELLULAR_STIMULUS | Any process that results in a change in state or activity of a cell or an organism (in terms of movement, secretion, enzyme production, gene expression, etc.) as a result of an extracellular stimulus. [GOC:go_curators] | 9 | 2.66E-11 | 9.55E-09 |
| GO_REGULATION_OF_TRANSPORT | Any process that modulates the frequency, rate or extent of the directed movement of substances (such as macromolecules, small molecules, ions) into, out of or within a cell, or between cells, by means of some agent such as a transporter or pore. [GOC:ai] | 13 | 2.92E-11 | 9.97E-09 |
| GO_NEGATIVE_REGULATION_OF_TRANSPORT | Any process that stops, prevents, or reduces the frequency, rate or extent of the directed movement of substances (such as macromolecules, small molecules, ions) into, out of or within a cell, or between cells, by means of some agent such as a transporter or pore. [GOC:ai] | 9 | 3.05E-11 | 9.97E-09 |
| GO_RESPONSE_TO_TOXIC_SUBSTANCE | Any process that results in a change in state or activity of a cell or an organism (in terms of movement, secretion, enzyme production, gene expression, etc.) as a result of a toxic stimulus. [GOC:lr] | 9 | 3.31E-11 | 1.04E-08 |
| GO_SMALL_MOLECULE_METABOLIC_PROCESS | The chemical reactions and pathways involving small molecules, any low molecular weight, monomeric, non-encoded molecule. [GOC:curators, GOC:pde, GOC:vw] | 13 | 4.53E-11 | 1.32E-08 |
| GO_STEROL_HOMEOSTASIS | Any process involved in the maintenance of an internal steady state of sterol within an organism or cell. [GOC:BHF, GOC:rl] | 6 | 4.55E-11 | 1.32E-08 |
| GO_STEROL_TRANSPORT | The directed movement of sterols into, out of or within a cell, or between cells, by means of some agent such as a transporter or pore. Sterols are steroids with one or more hydroxyl groups and a hydrocarbon side-chain in the molecule. [GOC:ai] | 6 | 5.79E-11 | 1.61E-08 |
| GO_NEGATIVE_REGULATION_OF_MACROPHAGE_DERIVED_FOAM_CELL_DIFFERENTIATION | Any process that decreases the rate, frequency or extent of macrophage derived foam cell differentiation. Macrophage derived foam cell differentiation is the process in which a macrophage acquires the specialized features of a foam cell. A foam cell is a type of cell containing lipids in small vacuoles and typically seen in atherosclerotic lesions, as well as other conditions. [GOC:add, GOC:BHF, GOC:dph, GOC:tb] | 4 | 9.94E-11 | 2.67E-08 |
| GO_NEGATIVE_REGULATION_OF_LIPID_TRANSPORT | Any process that stops, prevents, or reduces the frequency, rate or extent of the directed movement of lipids into, out of or within a cell, or between cells, by means of some agent such as a transporter or pore. [GOC:mah] | 5 | 1.15E-10 | 2.99E-08 |
| GO_CELLULAR_RESPONSE_TO_BIOTIC_STIMULUS | Any process that results in a change in state or activity of a cell (in terms of movement, secretion, enzyme production, gene expression, etc.) as a result of a biotic stimulus, a stimulus caused or produced by a living organism. [GOC:mah] | 7 | 1.93E-10 | 4.83E-08 |
| GO_ORGANIC_HYDROXY_COMPOUND_TRANSPORT | The directed movement of an organic hydroxy compound (organic alcohol) into, out of or within a cell, or between cells, by means of some agent such as a transporter or pore. An organic hydroxy compound is an organic compound having at least one hydroxy group attached to a carbon atom. [GOC:ai] | 7 | 2.21E-10 | 5.38E-08 |
| GO_RESPONSE_TO_HORMONE | Any process that results in a change in state or activity of a cell or an organism (in terms of movement, secretion, enzyme production, gene expression, etc.) as a result of a hormone stimulus. [GOC:jl] | 10 | 2.96E-10 | 6.97E-08 |
| GO_LIPID_HOMEOSTASIS | Any process involved in the maintenance of an internal steady state of lipid within an organism or cell. [GOC:BHF, GOC:rl] | 6 | 6.52E-10 | 1.49E-07 |
| GO_HOMEOSTATIC_PROCESS | Any biological process involved in the maintenance of an internal steady state. [GOC:jl, ISBN:0395825172] | 12 | 7.29E-10 | 1.61E-07 |
| GO_REGULATION_OF_RESPONSE_TO_EXTERNAL_STIMULUS | Any process that modulates the frequency, rate or extent of a response to an external stimulus. [GOC:mah] | 10 | 9.72E-10 | 2.09E-07 |
| GO_STEROID_METABOLIC_PROCESS | The chemical reactions and pathways involving steroids, compounds with a 1,2,cyclopentanoperhydrophenanthrene nucleus. [ISBN:0198547684] | 7 | 1.40E-09 | 2.92E-07 |
| GO_MAINTENANCE_OF_LOCATION | Any process in which a cell, substance or cellular entity, such as a protein complex or organelle, is maintained in a location and prevented from moving elsewhere. [GOC:ai, GOC:dph, GOC:tb] | 7 | 1.61E-09 | 3.28E-07 |
| GO_POSITIVE_REGULATION_OF_TRANSCRIPTION_BY_RNA_POLYMERASE_II | Any process that activates or increases the frequency, rate or extent of transcription from an RNA polymerase II promoter. [GOC:go_curators, GOC:txnOH] | 10 | 1.79E-09 | 3.55E-07 |
| GO_RECEPTOR_BIOSYNTHETIC_PROCESS | The chemical reactions and pathways resulting in the formation of a receptor molecule, a macromolecule that undergoes combination with a hormone, neurotransmitter, drug or intracellular messenger to initiate a change in cell function. [GOC:mah] | 4 | 2.07E-09 | 3.99E-07 |
| GO_NEURON_DEATH | The process of cell death in a neuron. [GOC:BHF, GOC:mah] | 7 | 2.18E-09 | 4.10E-07 |
| GO_ALCOHOL_METABOLIC_PROCESS | The chemical reactions and pathways involving alcohols, any of a class of compounds containing one or more hydroxyl groups attached to a saturated carbon atom. [ISBN:0198506732] | 7 | 3.43E-09 | 6.16E-07 |
| GO_CELLULAR_RESPONSE_TO_ORGANIC_CYCLIC_COMPOUND | Any process that results in a change in state or activity of a cell (in terms of movement, secretion, enzyme production, gene expression, etc.) as a result of an organic cyclic compound stimulus. [GOC:mah] | 8 | 3.47E-09 | 6.16E-07 |
| GO_CELLULAR_RESPONSE_TO_LIPID | Any process that results in a change in state or activity of a cell (in terms of movement, secretion, enzyme production, gene expression, etc.) as a result of a lipid stimulus. [GOC:mah] | 8 | 3.52E-09 | 6.16E-07 |
| GO_REGULATION_OF_MACROPHAGE_DERIVED_FOAM_CELL_DIFFERENTIATION | Any process that modulates the rate, frequency or extent of macrophage derived foam cell differentiation. Macrophage derived foam cell differentiation is the process in which a macrophage acquires the specialized features of a foam cell. A foam cell is a type of cell containing lipids in small vacuoles and typically seen in atherosclerotic lesions, as well as other conditions. [GOC:add, GOC:BHF, GOC:dph, GOC:tb] | 4 | 3.78E-09 | 6.47E-07 |
| GO_NEGATIVE_REGULATION_OF_MULTICELLULAR_ORGANISMAL_PROCESS | Any process that stops, prevents, or reduces the frequency, rate or extent of an organismal process, the processes pertinent to the function of an organism above the cellular level; includes the integrated processes of tissues and organs. [GOC:ai] | 10 | 4.48E-09 | 7.49E-07 |
| GO_POSITIVE_REGULATION_OF_TRANSPORT | Any process that activates or increases the frequency, rate or extent of the directed movement of substances (such as macromolecules, small molecules, ions) into, out of or within a cell, or between cells, by means of some agent such as a transporter or pore. [GOC:ai] | 9 | 6.49E-09 | 1.04E-06 |
| GO_REGULATION_OF_LIPID_METABOLIC_PROCESS | Any process that modulates the frequency, rate or extent of the chemical reactions and pathways involving lipids. [GOC:go_curators] | 7 | 6.54E-09 | 1.04E-06 |
| GO_REGULATION_OF_CATABOLIC_PROCESS | Any process that modulates the frequency, rate, or extent of the chemical reactions and pathways resulting in the breakdown of substances. [GOC:go_curators] | 9 | 6.66E-09 | 1.04E-06 |
| GO_FOAM_CELL_DIFFERENTIATION | The process in which a relatively unspecialized cell acquires the specialized features of a foam cell. A foam cell is a type of cell containing lipids in small vacuoles and typically seen in atherosclerotic lesions, as well as other conditions. [GOC:add, GOC:BHF, GOC:dph, GOC:tb] | 4 | 8.11E-09 | 1.25E-06 |
| GO_REGULATION_OF_SMALL_MOLECULE_METABOLIC_PROCESS | Any process that modulates the rate, frequency or extent of a small molecule metabolic process. [GOC:vw] | 7 | 9.19E-09 | 1.38E-06 |
| GO_CELLULAR_KETONE_METABOLIC_PROCESS | The chemical reactions and pathways involving any of a class of organic compounds that contain the carbonyl group, CO, and in which the carbonyl group is bonded only to carbon atoms, as carried out by individual cells. The general formula for a ketone is RCOR, where R and R are alkyl or aryl groups. [GOC:jl, ISBN:0787650153] | 6 | 1.09E-08 | 1.61E-06 |
| GO_SMALL_MOLECULE_BIOSYNTHETIC_PROCESS | The chemical reactions and pathways resulting in the formation of small molecules, any low molecular weight, monomeric, non-encoded molecule. [GOC:curators, GOC:pde, GOC:vw] | 8 | 1.24E-08 | 1.75E-06 |
| GO_REGULATION_OF_CHOLESTEROL_EFFLUX | Any process that modulates the frequency, rate or extent of cholesterol efflux. Cholesterol efflux is the directed movement of cholesterol, cholest-5-en-3-beta-ol, out of a cell or organelle. [GOC:BHF, GOC:dph, GOC:tb] | 4 | 1.26E-08 | 1.75E-06 |
| GO_REGULATION_OF_DIGESTIVE_SYSTEM_PROCESS | Any process that modulates the frequency, rate or extent of a digestive system process, a physical, chemical, or biochemical process carried out by living organisms to break down ingested nutrients into components that may be easily absorbed and directed into metabolism. [GOC:jl] | 4 | 1.26E-08 | 1.75E-06 |
| GO_POSITIVE_REGULATION_OF_CELL_DEATH | Any process that increases the rate or frequency of cell death. Cell death is the specific activation or halting of processes within a cell so that its vital functions markedly cease, rather than simply deteriorating gradually over time, which culminates in cell death. [GOC:dph, GOC:tb] | 8 | 1.44E-08 | 1.97E-06 |
| GO_NEGATIVE_REGULATION_OF_IMMUNE_SYSTEM_PROCESS | Any process that stops, prevents, or reduces the frequency, rate, or extent of an immune system process. [GOC:add] | 7 | 1.83E-08 | 2.46E-06 |
| GO_INFLAMMATORY_RESPONSE | The immediate defensive reaction (by vertebrate tissue) to infection or injury caused by chemical or physical agents. The process is characterized by local vasodilation, extravasation of plasma into intercellular spaces and accumulation of white blood cells and macrophages. [GO_REF:0000022, GOC:mtg_15nov05, ISBN:0198506732] | 8 | 1.92E-08 | 2.53E-06 |
| GO_REGULATION_OF_ESTABLISHMENT_OF_PROTEIN_LOCALIZATION | Any process that modulates the frequency, rate or extent of the directed movement of a protein to a specific location. [GOC:BHF, GOC:mah] | 8 | 1.96E-08 | 2.54E-06 |
| GO_DNA_TOPOLOGICAL_CHANGE | The process in which a transformation is induced in the topological structure of a double-stranded DNA helix, resulting in a change in linking number. [ISBN:071673706X, ISBN:0935702490] | 3 | 2.04E-08 | 2.61E-06 |
| GO_REGULATION_OF_RESPONSE_TO_STRESS | Any process that modulates the frequency, rate or extent of a response to stress. Response to stress is a change in state or activity of a cell or an organism (in terms of movement, secretion, enzyme production, gene expression, etc.) as a result of a disturbance in organismal or cellular homeostasis, usually, but not necessarily, exogenous (e.g. temperature, humidity, ionizing radiation). [GOC:dhl] | 10 | 2.21E-08 | 2.74E-06 |
| GO_RESPONSE_TO_NITROGEN_COMPOUND | Any process that results in a change in state or activity of a cell or an organism (in terms of movement, secretion, enzyme production, gene expression, etc.) as a result of a nitrogen compound stimulus. [GOC:pr, GOC:TermGenie] | 9 | 2.22E-08 | 2.74E-06 |
| GO_RESPONSE_TO_BIOTIC_STIMULUS | Any process that results in a change in state or activity of a cell or an organism (in terms of movement, secretion, enzyme production, gene expression, etc.) as a result of a biotic stimulus, a stimulus caused or produced by a living organism. [GOC:hb] | 10 | 2.32E-08 | 2.82E-06 |
| GO_RESPONSE_TO_ESTRADIOL | Any process that results in a change in state or activity of a cell or an organism (in terms of movement, secretion, enzyme production, gene expression, etc.) as a result of stimulus by estradiol, a C18 steroid hormone hydroxylated at C3 and C17 that acts as a potent estrogen. [GOC:mah, ISBN:0911910123] | 5 | 2.50E-08 | 2.99E-06 |
| GO_RHYTHMIC_PROCESS | Any process pertinent to the generation and maintenance of rhythms in the physiology of an organism. [GOC:jid] | 6 | 2.94E-08 | 3.46E-06 |
| GO_REGULATION_OF_CELLULAR_CATABOLIC_PROCESS | Any process that modulates the frequency, rate or extent of the chemical reactions and pathways resulting in the breakdown of substances, carried out by individual cells. [GOC:mah] | 8 | 3.63E-08 | 4.20E-06 |
| GO_ORGANIC_HYDROXY_COMPOUND_METABOLIC_PROCESS | The chemical reactions and pathways involving organic hydroxy compound. [GOC:pr, GOC:TermGenie] | 7 | 4.41E-08 | 5.03E-06 |
| GO_RESPONSE_TO_ABIOTIC_STIMULUS | Any process that results in a change in state or activity of a cell or an organism (in terms of movement, secretion, enzyme production, gene expression, etc.) as a result of an abiotic (not derived from living organisms) stimulus. [GOC:hb] | 9 | 4.59E-08 | 5.15E-06 |
| GO_NEGATIVE_REGULATION_OF_RESPONSE_TO_STIMULUS | Any process that stops, prevents, or reduces the frequency, rate or extent of a response to a stimulus. Response to stimulus is a change in state or activity of a cell or an organism (in terms of movement, secretion, enzyme production, gene expression, etc.) as a result of a stimulus. [GOC:jid] | 10 | 5.08E-08 | 5.63E-06 |
| GO_RESPONSE_TO_INORGANIC_SUBSTANCE | Any process that results in a change in state or activity of a cell or an organism (in terms of movement, secretion, enzyme production, gene expression, etc.) as a result of an inorganic substance stimulus. [GOC:sm] | 7 | 5.23E-08 | 5.68E-06 |
| GO_POSITIVE_REGULATION_OF_RNA_METABOLIC_PROCESS | Any process that activates or increases the frequency, rate or extent of the chemical reactions and pathways involving RNA. [GOC:ai] | 10 | 5.28E-08 | 5.68E-06 |
| GO_RESPONSE_TO_ANTIBIOTIC | Any process that results in a change in state or activity of a cell or an organism (in terms of movement, secretion, enzyme production, gene expression, etc.) as a result of an antibiotic stimulus. An antibiotic is a chemical substance produced by a microorganism which has the capacity to inhibit the growth of or to kill other microorganisms. [GOC:ai, GOC:ef] | 6 | 6.04E-08 | 6.41E-06 |
| GO_REGULATION_OF_CELL_DEATH | Any process that modulates the rate or frequency of cell death. Cell death is the specific activation or halting of processes within a cell so that its vital functions markedly cease, rather than simply deteriorating gradually over time, which culminates in cell death. [GOC:dph, GOC:tb] | 10 | 6.49E-08 | 6.79E-06 |
| GO_DEFENSE_RESPONSE | Reactions, triggered in response to the presence of a foreign body or the occurrence of an injury, which result in restriction of damage to the organism attacked or prevention/recovery from the infection caused by the attack. [GOC:go_curators] | 10 | 7.11E-08 | 7.33E-06 |
| GO_STEROL_METABOLIC_PROCESS | The chemical reactions and pathways involving sterols, steroids with one or more hydroxyl groups and a hydrocarbon side-chain in the molecule. [ISBN:0198547684] | 5 | 7.47E-08 | 7.60E-06 |
| GO_RESPONSE_TO_LAMINAR_FLUID_SHEAR_STRESS | Any process that results in a change in state or activity of a cell or an organism (in terms of movement, secretion, enzyme production, gene expression, etc.) as a result of a laminar fluid shear stress stimulus. Laminar fluid flow is the force acting on an object in a system where the fluid is moving across a solid surface in parallel layers. As an example, laminar shear stress can be seen where blood flows against the luminal side of blood vessel walls. [GOC:ecd] | 3 | 8.83E-08 | 8.86E-06 |
| GO_POSITIVE_REGULATION_OF_NUCLEOBASE_CONTAINING_COMPOUND_METABOLIC_PROCESS | Any cellular process that activates or increases the frequency, rate or extent of the chemical reactions and pathways involving nucleobases, nucleosides, nucleotides and nucleic acids. [GOC:go_curators] | 10 | 1.22E-07 | 1.21E-05 |
| GO_PEPTIDE_SECRETION | The controlled release of a peptide from a cell or a tissue. [GOC:add] | 7 | 1.29E-07 | 1.26E-05 |
| GO_POSITIVE_REGULATION_OF_CHOLESTEROL_EFFLUX | Any process that increases the frequency, rate or extent of cholesterol efflux. Cholesterol efflux is the directed movement of cholesterol, cholest-5-en-3-beta-ol, out of a cell or organelle. [GOC:BHF, GOC:dph, GOC:tb] | 3 | 1.36E-07 | 1.31E-05 |
| GO_RESPONSE_TO_STEROID_HORMONE | Any process that results in a change in state or activity of a cell or an organism (in terms of movement, secretion, enzyme production, gene expression, etc.) as a result of a steroid hormone stimulus. [GOC:go_curators] | 6 | 1.47E-07 | 1.40E-05 |
| GO_RECEPTOR_METABOLIC_PROCESS | The chemical reactions and pathways involving a receptor molecule, a macromolecule that undergoes combination with a hormone, neurotransmitter, drug or intracellular messenger to initiate a change in cell function. [GOC:jl] | 5 | 1.57E-07 | 1.48E-05 |
| GO_REGULATION_OF_LIPID_BIOSYNTHETIC_PROCESS | Any process that modulates the frequency, rate or extent of the chemical reactions and pathways resulting in the formation of lipids. [GOC:ai] | 5 | 1.78E-07 | 1.65E-05 |
| GO_CELLULAR_RESPONSE_TO_ENDOGENOUS_STIMULUS | Any process that results in a change in state or activity of a cell (in terms of movement, secretion, enzyme production, gene expression, etc.) as a result of a stimulus arising within the organism. [GOC:mah] | 9 | 1.84E-07 | 1.68E-05 |
| GO_EMBRYO_DEVELOPMENT | The process whose specific outcome is the progression of an embryo from its formation until the end of its embryonic life stage. The end of the embryonic stage is organism-specific. For example, for mammals, the process would begin with zygote formation and end with birth. For insects, the process would begin at zygote formation and end with larval hatching. For plant zygotic embryos, this would be from zygote formation to the end of seed dormancy. For plant vegetative embryos, this would be from the initial determination of the cell or group of cells to form an embryo until the point when the embryo becomes independent of the parent plant. [GOC:go_curators, GOC:isa_complete, GOC:mtg_sensu] | 8 | 1.86E-07 | 1.68E-05 |
| GO_NEGATIVE_REGULATION_OF_CELL_DEATH | Any process that decreases the rate or frequency of cell death. Cell death is the specific activation or halting of processes within a cell so that its vital functions markedly cease, rather than simply deteriorating gradually over time, which culminates in cell death. [GOC:BHF, GOC:dph, GOC:tb] | 8 | 1.88E-07 | 1.68E-05 |
| GO_NEGATIVE_REGULATION_OF_DIGESTIVE_SYSTEM_PROCESS | Any process that decreases the frequency, rate or extent of a digestive system process, a physical, chemical, or biochemical process carried out by living organisms to break down ingested nutrients into components that may be easily absorbed and directed into metabolism. [GOC:dph, GOC:tb] | 3 | 1.98E-07 | 1.73E-05 |
| GO_REGULATION_OF_PROTEIN_LOCALIZATION | Any process that modulates the frequency, rate or extent of any process in which a protein is transported to, or maintained in, a specific location. [GOC:dph, GOC:mah, GOC:tb] | 8 | 1.98E-07 | 1.73E-05 |
| GO_CIRCADIAN_RHYTHM | Any biological process in an organism that recurs with a regularity of approximately 24 hours. [GOC:bf, GOC:go_curators] | 5 | 2.11E-07 | 1.82E-05 |
| GO_NEGATIVE_REGULATION_OF_DEVELOPMENTAL_PROCESS | Any process that stops, prevents or reduces the rate or extent of development, the biological process whose specific outcome is the progression of an organism over time from an initial condition (e.g. a zygote, or a young adult) to a later condition (e.g. a multicellular animal or an aged adult). [GOC:ai] | 8 | 2.13E-07 | 1.82E-05 |
| GO_POSITIVE_REGULATION_OF_CELLULAR_BIOSYNTHETIC_PROCESS | Any process that activates or increases the frequency, rate or extent of the chemical reactions and pathways resulting in the formation of substances, carried out by individual cells. [GOC:mah] | 10 | 2.16E-07 | 1.83E-05 |
| GO_POSITIVE_REGULATION_OF_LIPID_LOCALIZATION | Any process that activates or increases the frequency, rate or extent of lipid localization. [GO_REF:0000058, GOC:TermGenie, PMID:17564681] | 4 | 2.36E-07 | 1.97E-05 |
| GO_CELLULAR_LIPID_METABOLIC_PROCESS | The chemical reactions and pathways involving lipids, as carried out by individual cells. [GOC:jl] | 8 | 2.41E-07 | 1.99E-05 |
| GO_LIPID_BIOSYNTHETIC_PROCESS | The chemical reactions and pathways resulting in the formation of lipids, compounds soluble in an organic solvent but not, or sparingly, in an aqueous solvent. [GOC:go_curators] | 7 | 2.58E-07 | 2.11E-05 |
| GO_RESPONSE_TO_NUTRIENT | Any process that results in a change in state or activity of a cell or an organism (in terms of movement, secretion, enzyme production, gene expression, etc.) as a result of a nutrient stimulus. [GOC:go_curators] | 5 | 2.85E-07 | 2.31E-05 |
| GO_EXECUTION_PHASE_OF_APOPTOSIS | A stage of the apoptotic process that starts with the controlled breakdown of the cell through the action of effector caspases or other effector molecules (e.g. cathepsins, calpains etc.). Key steps of the execution phase are rounding-up of the cell, retraction of pseudopodes, reduction of cellular volume (pyknosis), chromatin condensation, nuclear fragmentation (karyorrhexis), plasma membrane blebbing and fragmentation of the cell into apoptotic bodies. When the execution phase is completed, the cell has died. [GOC:mtg_apoptosis, PMID:21760595] | 4 | 3.00E-07 | 2.40E-05 |
| GO_RESPONSE_TO_REACTIVE_OXYGEN_SPECIES | Any process that results in a change in state or activity of a cell or an organism (in terms of movement, secretion, enzyme production, gene expression, etc.) as a result of a reactive oxygen species stimulus. Reactive oxygen species include singlet oxygen, superoxide, and oxygen free radicals. [GOC:krc] | 5 | 3.71E-07 | 2.92E-05 |
| GO_NEGATIVE_REGULATION_OF_CELL_DIFFERENTIATION | Any process that stops, prevents, or reduces the frequency, rate or extent of cell differentiation. [GOC:go_curators] | 7 | 3.73E-07 | 2.92E-05 |
| GO_HORMONE_METABOLIC_PROCESS | The chemical reactions and pathways involving any hormone, naturally occurring substances secreted by specialized cells that affects the metabolism or behavior of other cells possessing functional receptors for the hormone. [GOC:jl] | 5 | 4.04E-07 | 3.13E-05 |
| GO_POSITIVE_REGULATION_OF_ESTABLISHMENT_OF_PROTEIN_LOCALIZATION | Any process that activates or increases the frequency, rate or extent of establishment of protein localization. [GO_REF:0000058, GOC:TermGenie, PMID:22761445] | 6 | 4.12E-07 | 3.17E-05 |
| GO_MACROPHAGE_ACTIVATION | A change in morphology and behavior of a macrophage resulting from exposure to a cytokine, chemokine, cellular ligand, or soluble factor. [GOC:mgi_curators, ISBN:0781735149, PMID:14506301] | 4 | 4.45E-07 | 3.35E-05 |
| GO_POSITIVE_REGULATION_OF_NEURON_DEATH | Any process that activates or increases the frequency, rate or extent of neuron death. [GOC:rph, GOC:TermGenie] | 4 | 4.45E-07 | 3.35E-05 |

**Supplementary Table S3. List of molecular functions**

| **Molecular functions** | **Description** | **Genes in Overlap** | **p-value** | **FDR q-value** |
| --- | --- | --- | --- | --- |
| GO_REGULATORY_REGION_NUCLEIC_ACID_BINDING | Interacting selectively and non-covalently with a nucleic acid region that regulates a nucleic acid-based process. Such processes include transcription, DNA replication, and DNA repair. [GOC:txnOH] | 8 | 1.10E-07 | 1.82E-04 |
| GO_CYTOKINE_ACTIVITY | Functions to control the survival, growth, differentiation and effector function of tissues and cells. [ISBN:0198599471] | 5 | 2.79E-07 | 1.82E-04 |
| GO_STEROL_TRANSPORTER_ACTIVITY | Enables the directed movement of sterols into, out of or within a cell, or between cells. Sterol are steroids with one or more hydroxyl groups and a hydrocarbon side-chain in the molecule. [GOC:ai] | 3 | 3.72E-07 | 1.82E-04 |
| GO_SIGNALING_RECEPTOR_BINDING | Interacting selectively and non-covalently with one or more specific sites on a receptor molecule, a macromolecule that undergoes combination with a hormone, neurotransmitter, drug or intracellular messenger to initiate a change in cell function. [GOC:bf, GOC:ceb, ISBN:0198506732] | 9 | 4.37E-07 | 1.82E-04 |
| GO_CYTOKINE_RECEPTOR_BINDING | Interacting selectively and non-covalently with a cytokine receptor. [GOC:mah, GOC:vw] | 5 | 9.88E-07 | 3.28E-04 |
| GO_LIPID_TRANSPORTER_ACTIVITY | Enables the directed movement of lipids into, out of or within a cell, or between cells. [GOC:ai] | 4 | 1.24E-06 | 3.44E-04 |
| GO_DOUBLE_STRANDED_DNA_BINDING | Interacting selectively and non-covalently with double-stranded DNA. [GOC:elh, GOC:vw] | 7 | 2.23E-06 | 4.72E-04 |
| GO_CIS_REGULATORY_REGION_BINDING | Interacting selectively and non-covalently with a specific upstream regulatory DNA sequence (transcription factor recognition sequence or binding site) located in cis relative to the transcription start site (i.e., on the same strand of DNA) and which enhances transcription from that promoter. [GOC:sart, GOC:txnOH, SO:0000165] | 6 | 2.27E-06 | 4.72E-04 |
| GO_LIPID_TRANSFER_ACTIVITY | Removes a lipid from a membrane or a monolayer lipid particle, transports it through the aqueous phase while protected in a hydrophobic pocket, and brings it to an acceptor membrane or lipid particle. [GOC:krc, PMID:20823909, PMID:24220498, PMID:25797198] | 3 | 2.75E-06 | 5.08E-04 |
| GO_TUMOR_NECROSIS_FACTOR_RECEPTOR_SUPERFAMILY_BINDING | Interacting selectively and non-covalently with any member of the tumor necrosis factor receptor superfamily. [GOC:add] | 3 | 3.40E-06 | 5.63E-04 |
| GO_LIGAND_ACTIVATED_TRANSCRIPTION_FACTOR_ACTIVITY | A DNA-binding transcription factor activity that is directly regulated by binding of a ligand to the protein with this activity. Examples include the lac and trp repressors in E.coli and many steroid hormone receptors. [GOC:dos, http://www.ecocyc.org/ECOLI/NEW-IMAGE?object=BC-3.1.2.3] | 3 | 3.88E-06 | 5.63E-04 |
| GO_SUPERCOILED_DNA_BINDING | Interacting selectively and non-covalently with supercoiled DNA. For example, during replication and transcription, template DNA is negatively supercoiled in the receding downstream DNA and positively supercoiled in the approaching downstream DNA. [GOC:pr, GOC:rph, PMID:20723754, PMID:21345933, Wikipedia:DNA_supercoil] | 2 | 4.06E-06 | 5.63E-04 |
| GO_DNA_TOPOISOMERASE_ACTIVITY | Catalysis of the transient cleavage and passage of individual DNA strands or double helices through one another, resulting a topological transformation in double-stranded DNA. [GOC:mah, PMID:8811192] | 2 | 6.09E-06 | 7.79E-04 |
| GO_STEROID_HORMONE_RECEPTOR_ACTIVITY | Combining with a steroid hormone and transmitting the signal within the cell to initiate a change in cell activity or function. [GOC:signaling, PMID:14708019] | 3 | 6.60E-06 | 7.84E-04 |
| GO_DRUG_BINDING | Interacting selectively and non-covalently with a drug, any naturally occurring or synthetic substance, other than a nutrient, that, when administered or applied to an organism, affects the structure or functioning of the organism; in particular, any such substance used in the diagnosis, prevention, or treatment of disease. [GOC:jl, ISBN:0198506732] | 8 | 8.84E-06 | 9.80E-04 |
| GO_ESTROGEN_16_ALPHA_HYDROXYLASE_ACTI | Catalysis of the reaction: estrogen + donor-H2 + O2 = 16-alpha-hydroxyestrogen + H2O. [GOC:BHF] | 2 | 1.14E-05 | 1.16E-03 |
| GO_NUCLEAR_RECEPTOR_TRANSCRIPTION_COACTIVATOR_ACTIVITY | The function of a transcription cofactor that activates transcription in conjuction with a ligand-dependent nuclear receptor from a RNA polymerase II promoter; does not bind DNA itself. [GOC:dph, GOC:tb] | 3 | 1.19E-05 | 1.16E-03 |
| GO_PROTEIN_DIMERIZATION_ACTIVITY | The formation of a protein dimer, a macromolecular structure consists of two noncovalently associated identical or nonidentical subunits. [ISBN:0198506732] | 7 | 1.52E-05 | 1.40E-03 |
| GO_CYSTEINE_TYPE_ENDOPEPTIDASE_ACTIVITY_INVOLVED_IN_APOPTOTIC_SIGNALING_PATHWAY | Catalysis of the hydrolysis of internal, alpha-peptide bonds in a polypeptide chain by a mechanism in which the sulfhydryl group of a cysteine residue at the active center acts as a nucleophile, and contributing to the apoptotic signaling pathway. [GOC:mtg_apoptosis, http://en.wikipedia.org/wiki/Caspase, PMID:11717445] | 2 | 1.82E-05 | 1.46E-03 |
| GO_OXIDOREDUCTASE_ACTIVITY_ACTING_ON_CH_OR_CH2_GROUPS | Catalysis of an oxidation-reduction (redox) reaction in which a CH2 group acts as a hydrogen or electron donor and reduces a hydrogen or electron acceptor. [GOC:ai] | 2 | 1.82E-05 | 1.46E-03 |
| GO_SEQUENCE_SPECIFIC_DOUBLE_STRANDED_DNA_BINDING | Interacting selectively and non-covalently with double-stranded DNA of a specific nucleotide composition, e.g. GC-rich DNA binding, or with a specific sequence motif or type of DNA, e.g. promotor binding or rDNA binding. [GOC:dos, GOC:sl] | 6 | 1.84E-05 | 1.46E-03 |
| GO_RECEPTOR_REGULATOR_ACTIVITY | The function of interacting (directly or indirectly) with receptors such that the proportion of receptors in the active form is changed. [GOC:ceb] | 5 | 2.06E-05 | 1.56E-03 |
| GO_STEROID_BINDING | Interacting selectively and non-covalently with a steroid, any of a large group of substances that have in common a ring system based on 1,2-cyclopentanoperhydrophenanthrene. [GOC:jl, ISBN:0198506732] | 3 | 3.14E-05 | 2.27E-03 |
| GO_CYSTEINE_TYPE_ENDOPEPTIDASE_ACTIVITY_INVOLVED_IN_APOPTOTIC_PROCESS | Catalysis of the hydrolysis of internal, alpha-peptide bonds in a polypeptide chain by a mechanism in which the sulfhydryl group of a cysteine residue at the active center acts as a nucleophile, and contributing to the apoptotic process. [GOC:mtg_apoptosis] | 2 | 4.25E-05 | 2.94E-03 |
| GO_DEATH_RECEPTOR_BINDING | Interacting selectively and non-covalently with any member of the death receptor (DR) family. The DR family falls within the tumor necrosis factor receptor superfamily and is characterized by a cytoplasmic region of ~80 residues termed the death domain (DD). [GOC:ceb, GOC:rl, PMID:15654015] | 2 | 4.85E-05 | 3.23E-03 |
| GO_PRIMARY_ACTIVE_TRANSMEMBRANE_TRANSPORTER_ACTIVITY | Enables the transfer of a solute from one side of a membrane to the other, up the solute's concentration gradient, by binding the solute and undergoing a series of conformational changes. Transport works equally well in either direction and is powered by a primary energy source, directly using ATP. Primary energy sources known to be coupled to transport are chemical, electrical and solar sources. [GOC:mtg_transport, ISBN:0815340729, TC:3.-.- | 3 | 5.16E-05 | 3.30E-03 |
| GO_TRANSCRIPTION_COACTIVATOR_ACTIVITY | A protein or a member of a complex that interacts specifically and non-covalently with a DNA-bound DNA-binding transcription factor to activate the transcription of specific genes. Coactivators often act by altering chromatin structure and modifications. For example, one class of transcription coregulators modifies chromatin structure through covalent modification of histones. A second ATP-dependent class modifies the conformation of chromatin. Another type of coregulator activity is the bridging of a DNA-binding transcription factor to the basal transcription machinery. The Mediator complex, which bridges transcription factors and RNA polymerase, is also a transcription coactivator. [GOC:txnOH-2018, PMID:10213677, PMID:16858867] | 4 | 5.40E-05 | 3.33E-03 |
| GO_TRANSCRIPTION_FACTOR_BINDING | Interacting selectively and non-covalently with a transcription factor, a protein required to initiate or regulate transcription. [ISBN:0198506732] | 5 | 6.16E-05 | 3.66E-03 |
| GO_ACTIVE_TRANSMEMBRANE_TRANSPORTER_ACTIVITY | Enables the transfer of a specific substance or related group of substances from one side of a membrane to the other, up the solute's concentration gradient. The transporter binds the solute and undergoes a series of conformational changes. Transport works equally well in either direction. [GOC:mtg_transport, ISBN:0815340729] | 4 | 7.13E-05 | 4.09E-03 |
| GO_DNA_BINDING_BENDING | The activity of binding selectively and non-covalently to and distorting the original structure of DNA, typically a straight helix, into a bend, or increasing the bend if the original structure was intrinsically bent due to its sequence. [GOC:krc, GOC:vw, PMID:10710711, PMID:19037758] | 2 | 7.67E-05 | 4.25E-03 |
| GO_DNA_BINDING_TRANSCRIPTION_FACTOR_BINDING | Interacting selectively and non-covalently with a DNA-binding transcription factor, a protein that interacts with a specific DNA sequence (sometimes referred to as a motif) within the regulatory region of a gene to modulate transcription. [GOC:txnOH-2018] | 4 | 8.05E-05 | 4.32E-03 |
| GO_SEQUENCE_SPECIFIC_DNA_BINDING | Interacting selectively and non-covalently with DNA of a specific nucleotide composition, e.g. GC-rich DNA binding, or with a specific sequence motif or type of DNA e.g. promotor binding or rDNA binding. [GOC:jl] | 6 | 9.29E-05 | 4.81E-03 |
| GO_LIPID_BINDING | Interacting selectively and non-covalently with a lipid. [GOC:ai] | 5 | 9.55E-05 | 4.81E-03 |
| GO_OXIDOREDUCTASE_ACTIVITY | Catalysis of an oxidation-reduction (redox) reaction, a reversible chemical reaction in which the oxidation state of an atom or atoms within a molecule is altered. One substrate acts as a hydrogen or electron donor and becomes oxidized, while the other acts as hydrogen or electron acceptor and becomes reduced. [GOC:go_curators] | 5 | 1.08E-04 | 5.27E-03 |
| GO_AROMATASE_ACTIVITY | Catalysis of the reduction of an aliphatic ring to yield an aromatic ring. [GOC:cb] | 2 | 1.41E-04 | 6.72E-03 |
| GO_ATPASE_ACTIVITY | Catalysis of the reaction: ATP + H2O = ADP + phosphate + 2 H+. May or may not be coupled to another reaction. [EC:3.6.1.3, GOC:jl] | 4 | 1.75E-04 | 8.07E-03 |
| GO_TUMOR_NECROSIS_FACTOR_RECEPTOR_BINDING | Interacting selectively and non-covalently with the tumor necrosis factor receptor. [GOC:ai] | 2 | 1.87E-04 | 8.40E-03 |
| GO_OXIDOREDUCTASE_ACTIVITY_ACTING_ON_PAIRED_DONORS_WITH_INCORPORATION_OR_REDUCTION_OF_MOLECULAR_OXYGEN_REDUCED_FLAVIN_OR_FLAVOPROTEIN_AS_ONE_DONOR_AND_INCORPORATION_OF_ONE_ATOM_OF_OXYGEN | Catalysis of an oxidation-reduction (redox) reaction in which hydrogen or electrons are transferred from reduced flavin or flavoprotein and one other donor, and one atom of oxygen is incorporated into one donor. [GOC:mah] | 2 | 2.53E-04 | 1.11E-02 |
| GO_STEROID_HYDROXYLASE_ACTIVITY | Catalysis of the formation of a hydroxyl group on a steroid by incorporation of oxygen from O2. [ISBN:0721662544] | 2 | 2.82E-04 | 1.20E-02 |
| GO_TRANSCRIPTION_COFACTOR_BINDING | Interacting selectively and non-covalently with a transcription cofactor, any protein involved in regulation of transcription via protein-protein interactions with transcription factors and other transcription regulatory proteins. Cofactors do not bind DNA directly, but rather mediate protein-protein interactions between regulatory transcription factors and the basal transcription machinery. [GOC:krc] | 2 | 3.61E-04 | 1.50E-02 |
| GO_TRANSCRIPTION_COREGULATOR_ACTIVITY | A protein or a member of a complex that interacts specifically and non-covalently with a DNA-bound DNA-binding transcription factor to either activate or repress the transcription of specific genes. Coregulators often act by altering chromatin structure and modifications. For example, one class of transcription coregulators modifies chromatin structure through covalent modification of histones. A second ATP-dependent class modifies the conformation of chromatin. A third class modulates interactions of DNA-binding transcription factor with other transcription coregulators. [GOC:txnOH-2018, PMID:10213677, PMID:16858867, PMID:24203923, PMID:25957681, Wikipedia:Transcription_coregulator] | 4 | 4.68E-04 | 1.90E-02 |
| GO_TRANSITION_METAL_ION_BINDING | Interacting selectively and non-covalently with a transition metal ions; a transition metal is an element whose atom has an incomplete d-subshell of extranuclear electrons, or which gives rise to a cation or cations with an incomplete d-subshell. Transition metals often have more than one valency state. Biologically relevant transition metals include vanadium, manganese, iron, copper, cobalt, nickel, molybdenum and silver. [ISBN:0198506732] | 5 | 5.84E-04 | 2.29E-02 |
| GO_STEROL_BINDING | Interacting selectively and non-covalently with a sterol, any steroid containing a hydroxy group in the 3 position, closely related to cholestan-3-ol. [GOC:mah] | 2 | 5.91E-04 | 2.29E-02 |
| GO_DNA_BINDING_TRANSCRIPTION_FACTOR_ACTIVITY_RNA_POLYMERASE_II_SPECIFIC | A protein or a member of a complex that interacts selectively and non-covalently with a specific DNA sequence (sometimes referred to as a motif) within the regulatory region of a RNA polymerase II-transcribed gene to modulate transcription. Regulatory regions include promoters (proximal and distal) and enhancers. Genes are transcriptional units. [GOC:txnOH-2018] | 5 | 6.14E-04 | 2.32E-02 |
| GO_TRANSCRIPTION_REGULATOR_ACTIVITY | A molecular function that controls the rate, timing and/or magnitude of transcription of genetic information. The function of transcriptional regulators is to modulate gene expression at the transcription step so that they are expressed in the right cell at the right time and in the right amount throughout the life of the cell and the organism. [GOC:pg, GOC:txnOH-2018, Wikipedia:Transcription_factor] | 6 | 6.75E-04 | 2.50E-02 |
| GO_PROTEIN_CONTAINING_COMPLEX_BINDING | Interacting selectively and non-covalently with a macromolecular complex. [GOC:jl] | 5 | 7.25E-04 | 2.62E-02 |
| GO_IDENTICAL_PROTEIN_BINDING | Interacting selectively and non-covalently with an identical protein or proteins. [GOC:jl] | 6 | 7.67E-04 | 2.71E-02 |
| GO_RNA_POLYMERASE_II_SPECIFIC_DNA_BINDING_TRANSCRIPTION_FACTOR_BINDING | Interacting selectively and non-covalently with a sequence-specific DNA binding RNA polymerase II transcription factor, any of the factors that interact selectively and non-covalently with a specific DNA sequence in order to modulate transcription. [GOC:dph, GOC:vw] | 3 | 7.91E-04 | 2.71E-02 |
| GO_TRANSFERASE_ACTIVITY_TRANSFERRING_GLYCOSYL_GROUPS | Catalysis of the transfer of a glycosyl group from one compound (donor) to another (acceptor). [GOC:jl, ISBN:0198506732] | 3 | 7.99E-04 | 2.71E-02 |
| GO_MOLECULAR_FUNCTION_REGULATOR | A molecular function that modulates the activity of a gene product or complex. Examples include enzyme regulators and channel regulators. [GOC:dos, GOC:pt] | 6 | 8.93E-04 | 2.97E-02 |
| GO_REPRESSING_TRANSCRIPTION_FACTOR_BINDING | Interacting selectively and non-covalently with a transcription repressor, any protein whose activity is required to prevent or downregulate transcription. [GOC:mah, GOC:txnOH] | 2 | 1.04E-03 | 3.39E-02 |
| GO_PROTEIN_DOMAIN_SPECIFIC_BINDING | Interacting selectively and non-covalently with a specific domain of a protein. [GOC:go_curators] | 4 | 1.09E-03 | 3.48E-02 |
| GO_ATPASE_BINDING | Interacting selectively and non-covalently with an ATPase, any enzyme that catalyzes the hydrolysis of ATP. [GOC:ai] | 2 | 1.25E-03 | 3.91E-02 |
| GO_ALCOHOL_BINDING | Interacting selectively and non-covalently with an alcohol, any of a class of alkyl compounds containing a hydroxyl group. [GOC:jl, ISBN:0198506732] | 2 | 1.37E-03 | 4.23E-02 |
| GO_DNA_BINDING_TRANSCRIPTION_FACTOR_ACTIVITY | A protein or a member of a complex that interacts selectively and non-covalently with a specific DNA sequence (sometimes referred to as a motif) within the regulatory region of a gene to modulate transcription. Regulatory regions include promoters (proximal and distal) and enhancers. Genes are transcriptional units, and include bacterial operons. [GOC:txnOH-2018] | 5 | 1.49E-03 | 4.51E-02 |

**Supplementary Table S4. List of cellular components**

| **Cellular components** | **Description** | **Genes in Overlap** | **p-value** | **FDR q-value** |
| --- | --- | --- | --- | --- |
| GO_NUCLEAR_CHROMOSOME | A chromosome that encodes the nuclear genome and is found in the nucleus of a eukaryotic cell during the cell cycle phases when the nucleus is intact. [GOC:dph, GOC:mah] | 8 | 9.32E-07 | 9.32E-04 |
| GO_NUCLEAR_OUTER_MEMBRANE_ENDOPLASMIC_RETICULUM_MEMBRANE_NETWORK | The continuous network of membranes encompassing the nuclear outer membrane and the endoplasmic reticulum membrane. [GOC:bf, GOC:jl, GOC:mah, GOC:mcc, GOC:pr, GOC:vw] | 7 | 4.69E-06 | 2.03E-03 |
| GO_ATP_BINDING_CASSETTE_ABC_TRANSPORTER_COMPLEX | A complex for the transport of metabolites into and out of the cell, typically comprised of four domains; two membrane-associated domains and two ATP-binding domains at the intracellular face of the membrane, that form a central pore through the plasma membrane. Each of the four core domains may be encoded as a separate polypeptide or the domains can be fused in any one of a number of ways into multidomain polypeptides. In Bacteria and Archaebacteria, ABC transporters also include substrate binding proteins to bind substrate external to the cytoplasm and deliver it to the transporter. [GOC:jl, GOC:mtg_sensu, PMID:11421269, PMID:15111107] | 2 | 6.09E-06 | 2.03E-03 |
| GO_CHROMOSOME | A structure composed of a very long molecule of DNA and associated proteins (e.g. histones) that carries hereditary information. [ISBN:0198547684] | 8 | 9.07E-06 | 2.27E-03 |
| GO_DEATH_INDUCING_SIGNALING_COMPLEX | A protein complex formed by the association of signaling proteins with a death receptor upon ligand binding. The complex includes procaspases and death domain-containing proteins in addition to the ligand-bound receptor, and may control the activation of caspases 8 and 10. [GOC:mtg_apoptosis, PMID:12628743, PMID:12655293, PMID:8521815] | 2 | 1.46E-05 | 2.92E-03 |
| GO_MEMBRANE_REGION | A membrane that is a part of a larger membrane. Examples include the apical region of the plasma membrane of an epithelial cell and the various regions of the endoplasmic reticulum membrane. [GOC:dos] | 4 | 5.87E-05 | 9.69E-03 |
| GO_PLASMA_MEMBRANE_PROTEIN_COMPLEX | Any protein complex that is part of the plasma membrane. [GOC:dos] | 5 | 6.79E-05 | 9.69E-03 |
| GO_ATPASE_DEPENDENT_TRANSMEMBRANE_TRANSPORT_COMPLEX | A transmembrane protein complex that functions in ATPase dependent active transport across a membrane. [GOC:dos] | 2 | 8.48E-05 | 1.06E-02 |
| GO_CHROMATIN | The ordered and organized complex of DNA, protein, and sometimes RNA, that forms the chromosome. [GOC:elh, PMID:20404130] | 6 | 1.10E-04 | 1.22E-02 |
| GO_MEMBRANE_PROTEIN_COMPLEX | Any protein complex that is part of a membrane. [GOC:dos] | 6 | 1.38E-04 | 1.38E-02 |
| GO_RNA_POLYMERASE_II_TRANSCRIPTION_FACTOR_COMPLEX | A transcription factor complex that acts at a regulatory region of a gene transcribed by RNA polymerase II. [GOC:tb] | 3 | 1.64E-04 | 1.49E-02 |
| GO_NUCLEAR_TRANSCRIPTION_FACTOR_COMPLEX | A protein complex, located in the nucleus, that is capable of associating with DNA by direct binding, or via other DNA-binding proteins or complexes, and regulating transcription. [GOC:jl] | 3 | 3.07E-04 | 2.56E-02 |
| GO_CELL_BODY | The portion of a cell bearing surface projections such as axons, dendrites, cilia, or flagella that includes the nucleus, but excludes all cell projections. [GOC:go_curators] | 4 | 4.68E-04 | 3.60E-02 |
| GO_WHOLE_MEMBRANE | Any lipid bilayer that completely encloses some structure, and all the proteins embedded in it or attached to it. Examples include the plasma membrane and most organelle membranes. [GOC:dos] | 6 | 5.83E-04 | 4.16E-02 |

**Supplementary Table S5. List of of KEGG pathways**

| **KEGG pathways** | **Genes in Overlap** | **p-value** | **FDR q-value** |
| --- | --- | --- | --- |
| Retinol metabolism | 4 | 8.64E-08 | 8.64E-06 |
| Metabolism of xenobiotics by cytochrome P450 | 4 | 1.24E-07 | 8.64E-06 |
| Drug metabolism - cytochrome P450 | 4 | 1.39E-07 | 8.64E-06 |
| Linoleic acid metabolism | 3 | 8.80E-07 | 4.09E-05 |
| ABC transporters | 3 | 3.17E-06 | 1.18E-04 |
| Drug metabolism - other enzymes | 3 | 4.97E-06 | 1.54E-04 |
| Steroid hormone biosynthesis | 3 | 6.25E-06 | 1.66E-04 |
| NOD-like receptor signaling pathway | 3 | 8.98E-06 | 2.09E-04 |
| RIG-I-like receptor signaling pathway | 3 | 1.35E-05 | 2.80E-04 |
| Apoptosis | 3 | 2.49E-05 | 4.63E-04 |
| Toll-like receptor signaling pathway | 3 | 4.01E-05 | 6.78E-04 |
| Ascorbate and aldarate metabolism | 2 | 1.21E-04 | 1.87E-03 |
| Pentose and glucuronate interconversions | 2 | 1.52E-04 | 2.17E-03 |
| Alzheimer's disease | 3 | 1.67E-04 | 2.17E-03 |
| Asthma | 2 | 1.75E-04 | 2.17E-03 |
| Allograft rejection | 2 | 2.67E-04 | 3.11E-03 |
| Porphyrin and chlorophyll metabolism | 2 | 3.28E-04 | 3.59E-03 |
| Starch and sucrose metabolism | 2 | 5.29E-04 | 5.38E-03 |
| Amyotrophic lateral sclerosis (ALS) | 2 | 5.49E-04 | 5.38E-03 |
| Cytokine-cytokine receptor interaction | 3 | 6.68E-04 | 6.21E-03 |
| Adipocytokine signaling pathway | 2 | 8.76E-04 | 7.11E-03 |
| Epithelial cell signaling in Helicobacter pylori infection | 2 | 9.03E-04 | 7.11E-03 |
| p53 signaling pathway | 2 | 9.03E-04 | 7.11E-03 |
| PPAR signaling pathway | 2 | 9.29E-04 | 7.11E-03 |
| Viral myocarditis | 2 | 9.56E-04 | 7.11E-03 |
| Leishmania infection | 2 | 1.01E-03 | 7.23E-03 |
| Pathways in cancer | 3 | 1.20E-03 | 8.29E-03 |
| T cell receptor signaling pathway | 2 | 2.25E-03 | 1.50E-02 |
| Natural killer cell mediated cytotoxicity | 2 | 3.59E-03 | 2.29E-02 |
| Systemic lupus erythematosus | 2 | 3.69E-03 | 2.29E-02 |
| Huntington's disease | 2 | 6.11E-03 | 3.66E-02 |
